# Synthesis and characterization of carbonyl functionalized organotellurium(iv) derivatives†

**DOI:** 10.1039/d4ra06023g

**Published:** 2024-11-08

**Authors:** Puspendra Singh, Mariya Khan, Andrew Duthie, Ray J. Butcher

**Affiliations:** a Department of Chemistry, Dr Shakuntala Misra National Rehabilitation University Lucknow 226017 India pushpendrasingh0612@gmail.com; b Department of Chemistry, University of Lucknow Lucknow 226007 India; c School of Life and Environmental Sciences, Deakin University Geelong 3217 Australia; d Department of Chemistry, Howard University Washington DC20059 USA

## Abstract

The current study focuses on synthesis and characterization of carbonyl functionalized unsymmetrical diorganotellurium(iv) dichlorides (1–5), dibromide (6), and their characterization by elemental analysis, ^1^H, ^13^C{^1^H}, and ^125^Te NMR spectroscopy. In addition to this, compounds 1, 4 and 5 were further confirmed *via* single-crystal X-ray studies. Reduction of all the dichlorides with Na_2_S_2_O_5_ affords labile tellurides, which decompose quickly even at room temperature into the more stable symmetric ditellurides, Ar_2_Te_2_. Mesityl fragments bearing organotellurium(iv) derivatives show separate ^1^H and ^13^C{^1^H} NMR signals for the *ortho* methyls and *meta* protons of the mesityl ring. Among the Te(iv) dichlorides, the observed O–H⋯O, C–H⋯O, C–H⋯Cl, Te⋯O and Te⋯Cl hydrogen and secondary bonding interactions are longer than Σ*r*_cov_ (sum of the covalent bond radii) and significantly shorter than Σ*r*_vdw_ (sum of the van der Waal radii) respectively. The linearity of the C–Te⋯O, C–H⋯O and C–H⋯Cl makes n → σ* orbital interaction possible.

## Abstract 2

Aryltellurium trichlorides, ArTeCl_3_ (Ar = C_6_H_5_, Ph; 4-Me-C_6_H_4_, *p*-Tol; 1-C_10_H_7_, 1-Nap; 2,4,6-Me_3_C_6_H_2_, Mes) react under mild conditions with acetylacetone/benzoylacetone to give carbonyl functionalized organotellurium(iv) derivatives.

## Graphical abstract

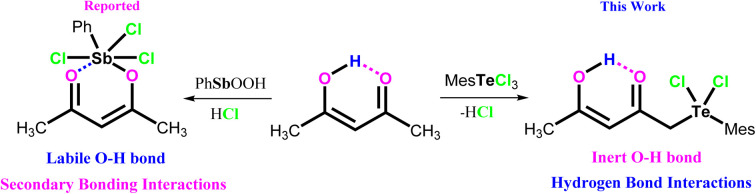

## 1 Introduction

**Chart 1:**
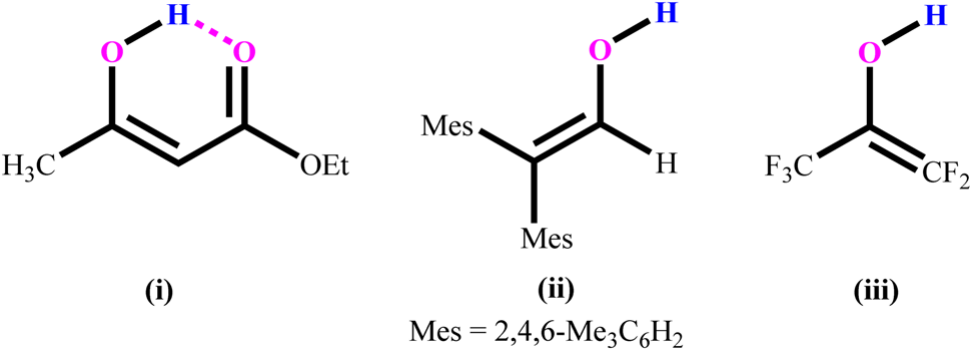
Three main types of the more stable enols.

The structure and reactivity of acetylacetone and benzoylacetone molecules is important in the creation of inter- and intramolecular hydrogen bonds in many fields of science,^1–7^ with the bonds playing an essential role in areas of chemistry ranging from biochemistry^8^ to crystal engineering,^9^ self-assembly of large pore zeolites^10^ and supramolecular chemistry^11–14^ to catalysis.^15–19^ The acetylacetone and benzoylacetone molecules can exist in keto and enol forms, generally the keto form is more thermodynamically stable than the enol form. Interestingly, it is observed that the enol form is predominant in acetylacetone and benzoylacetone, with percentages of 80.0 and 89.4% respectively.^20^ The following three main types of the more stable enol tautomer are present: (i) in which the enolic double bond is in conjugation with another double bond, (ii) a molecule bearing two or three bulky aryl groups, and (iii) a molecule bearing highly fluorinated enols (Chart 1).^20^

**Chart 2:**
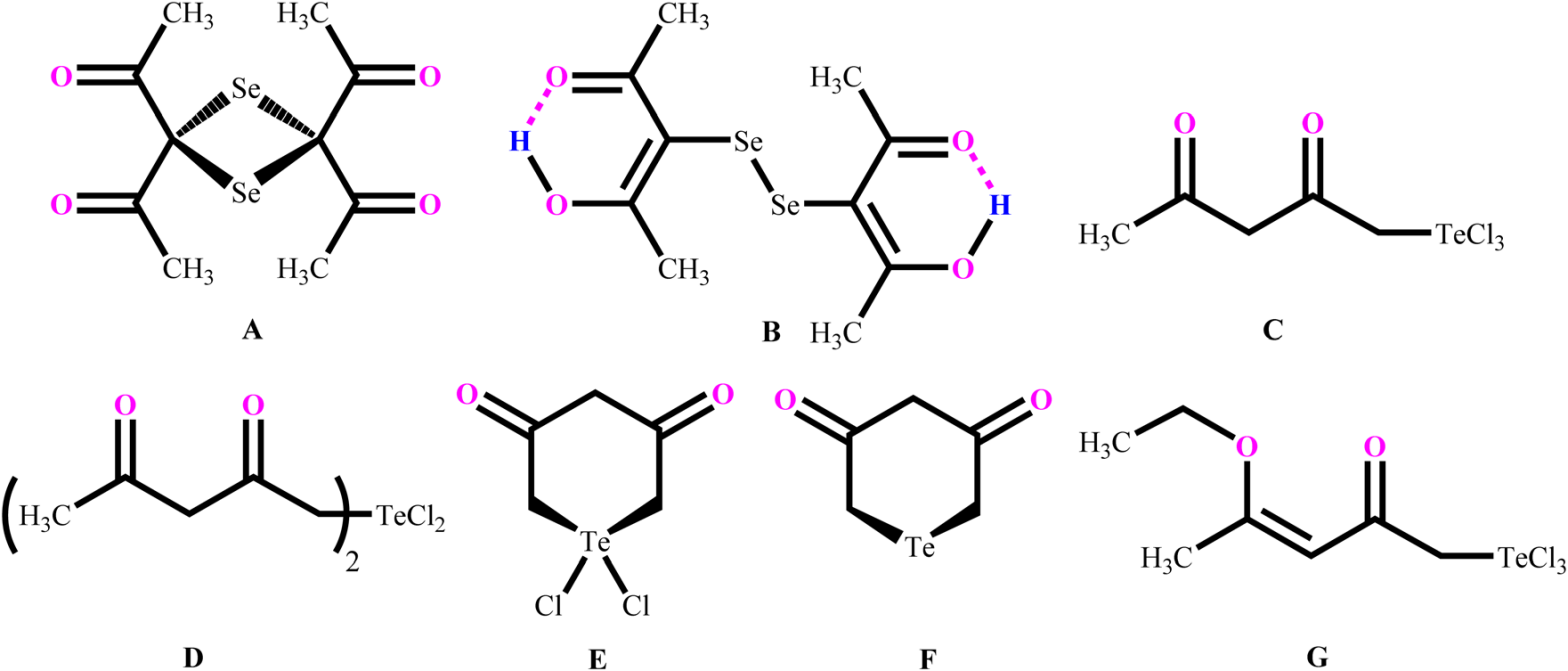
Reported organoselenium and organotellurium derivatives of acetylacetone.

Morgan and co-workers have reported chalcogen derivatives (A–G) of acetylacetone through the treatment of acetylacetone with SeCl_4_ and TeCl_4_ (Chart 2).^21^ Reaction of acetylacetone with SeCl_4_ can afford [(MeCO)_2_CSe]_2_ (A) as the major product. Reduction of A with hydroiodic acid can afford diselenide (B). Similarly, reaction of acetylacetone with TeCl_4_ (1 : 1) in chloroform affords the labile acetylacetonyltellurium trichloride [CH_3_C(O)CH_2_C(O)CH_2_]TeCl_3_ (C), with further addition of one equivalent of acetylacetone affording a mixture of the three derivatives: [CH_3_C(O)CH_2_C(O)CH_2_]_2_TeCl_2_ (D),
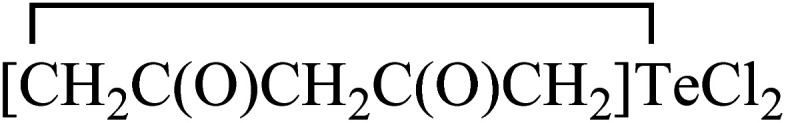
 (E) and 
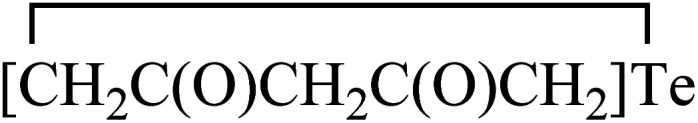
 (F).

Reaction of *in situ* generated molecule C with ethyl chloride can afford [CH_3_C(OEt)CHC(O)CH_2_]TeCl_3_ (G) in quantitative yield. Reaction of phenylstibonic acid with acetylacetone in the presence of HCl can afford trichloro(acetylacetonato)phenylantimony(v) as transparent needle-shaped crystals. Investigations with ^1^H-NMR and IR spectra revealed that only oxygen atoms of acetylacetone are covalently bonded with Sb atoms in the trichloro(acetylacetonato)phenylantimony(v).^22^

Similarly, methyl fragments bearing ketones can undergo electrophilic substitution reactions with aryltellurium trichlorides under mild conditions to obtain the aryl(acylmethyl)tellurium dichlorides Ar[RC(O)CH_2_]TeCl_2_ (Ar = phenyl, *p*-tolyl, *p*-anisyl, 1-naphthyl, mesityl; R = Me, *i*-Pr, *t*-Bu, mesityl).^23^ In the present study, we create the carbonyl functionalized organotellurium(iv) derivatives, Ar[RC(OH)CHC(O)CH_2_]TeCl_2_ (1–5) [Ar = phenyl, (Ph); *p*-tolyl, (*p*-tol) 1-napthyl, (1-Nap); and mesityl (Mes); R = methyl, phenyl] through the treatment of acetylacetone or benzoylacetone with aryltellurium trichlorides under mild conditions. In addition, Mes[CH_3_C(OH)CHC(O)CH_2_]TeBr_2_ (6) are also prepared through the metathetical reaction of 5 with NaBr in chloroform at room temperature.

## 2 Result and discussion

**Scheme 1:**
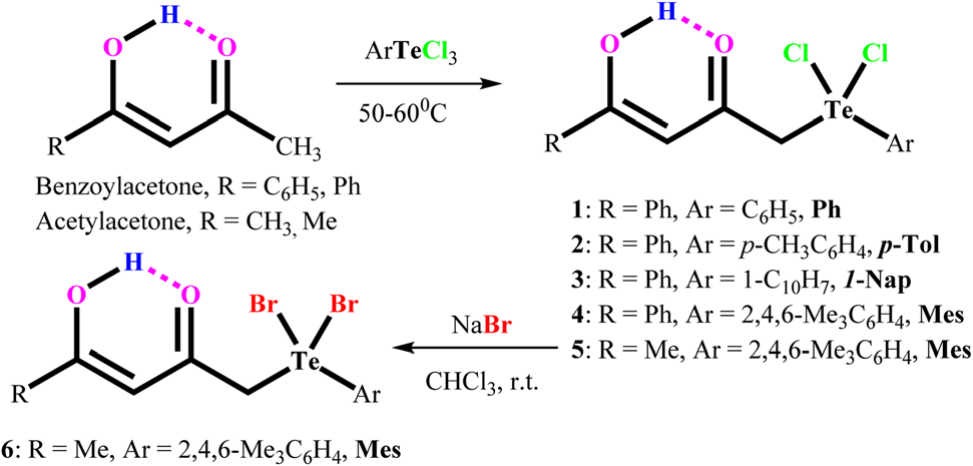
Development of carbonyl functionalized organotellurium(iv) derivatives.

Aryltellurium trichlorides react under mild conditions with acetylacetone/benzoylacetone to give proton responsive unsymmetrical diorganotellurium(iv) dichlorides, Ph[PhC(OH)CHC(O)CH_2_]TeCl_2_, (1); *p*-Tol[PhC(OH)CHC(O)CH_2_]TeCl_2_, (2); 1-Nap[PhC(OH)CHC(O)CH_2_]TeCl_2_, (3); Mes[PhC(OH)CHC(O)CH_2_]TeCl_2_, (4); and Mes[MeC(OH)CHC(O)CH_2_]TeCl_2_, (5) (Scheme 1). The aryltellurium trichlorides employed have two axial and one equatorial chlorine atoms along with the aryl fragment. Only the equatorial chlorine atom of the aryltellurium trichlorides participates in the reactions.^23^ We can conclude that aryl fragment have more *trans* directing effect than the chlorine atoms in the aryltellurium trichlorides. However, the reaction of 1-napthyltellurium triiodide with benzoylacetone did not take place, even at temperatures up to 60 °C. Similarly, decomposition takes place during reaction of 1-napthyltellurium tribromide, affording the bi-naphthyl product through aryl–aryl coupling.

**Scheme 2:**
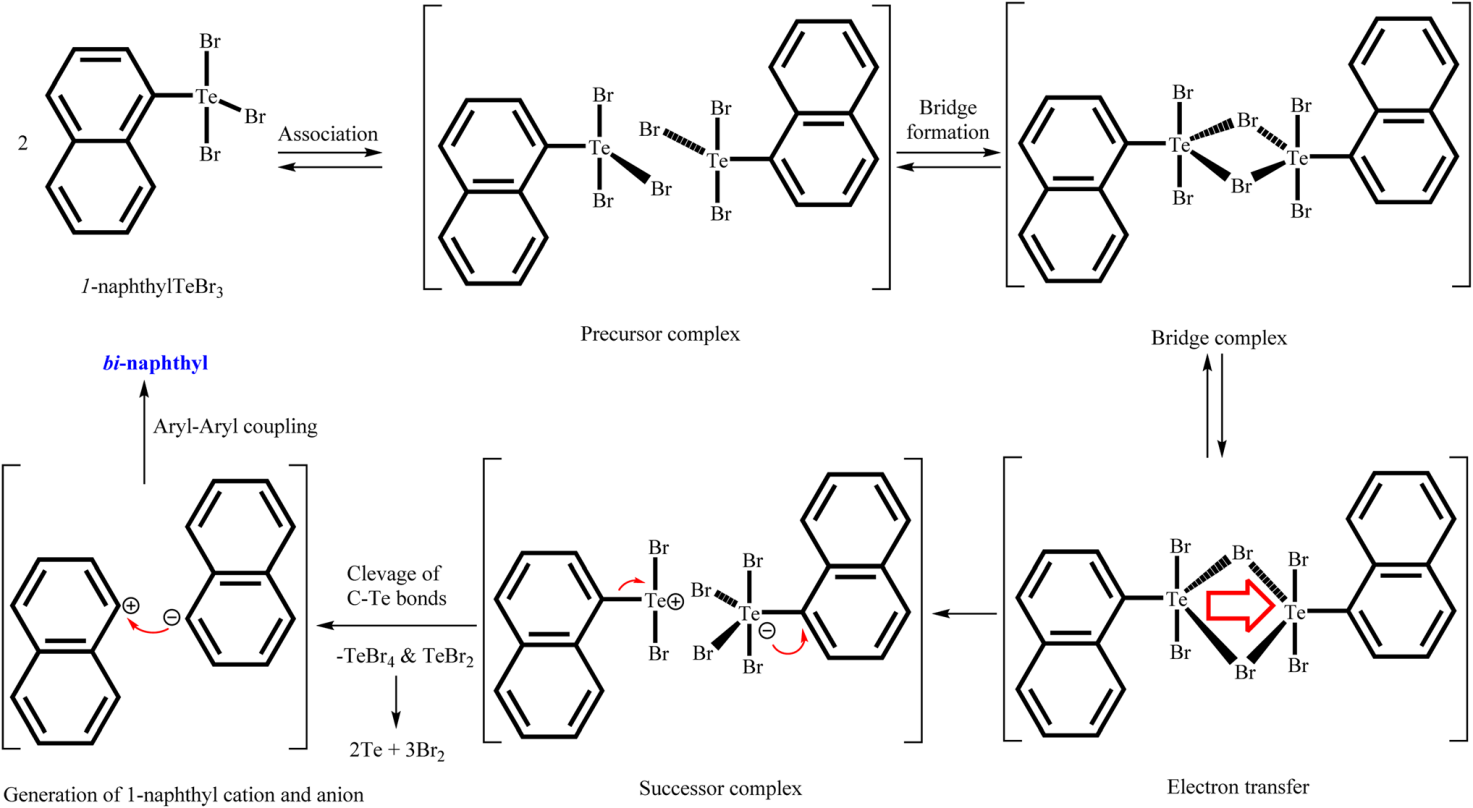
Plausible mechanism for aryl–aryl coupling.

Plausible mechanism for aryl–aryl coupling are shown in Scheme 2. The first two steps are the formation of a precursor complex and the formation of bridged binuclear intermediate. Subsequently electron transfer through the bridging ligand to give the successor complex, followed by dissociation to 1-napthyl cation, 1-napthyl anion, TeBr_4_ and TeBr_2_. Finally, 1-napthyl cation and anion undergo aryl–aryl coupling to give bi-naphthyl product. Simultaneously, during dissociation process also afforded two equivalent of elemental Te and three equivalent of elemental Br_2_ through decomposition of *in sittu* generated TeBr_4_ and TeBr_2_.

The dibromide Mes[PhC(OH)CHC(O)CH_2_]TeBr_2_, (6) can also be obtained with 95% yield from the corresponding dichlorides by metathesis with NaBr in chloroform at room temperature. Biphasic (H_2_O/CH_2_Cl_2_) reduction of dichlorides 1–5 with Na_2_S_2_O_5_ affords respective labile tellurides, which decompose quickly even at room temperature into the more stable symmetric ditellurides, Ar_2_Te_2_. The filtrate showed the presence of parent ketone (^1^H NMR).

All the proton responsive unsymmetrical diorganotellurium(iv) dihalides are crystalline solids that are soluble in common organic solvents. The ^1^H and ^13^C{^1^H} NMR spectra of the mesityltellurim(IV) derivatives are quite interesting. The restricted rotation of the mesityl fragment about the Te–C(mesityl) bond in 4, 5 and 6 is evidenced from their ^1^ H NMR spectra, which show separate signals for each of the *meta* ring protons and those of the two *ortho* methyl groups.^23,24^ All of the corresponding ^13^C{^1^H} NMR spectra consist of separate signals for each of the six ring and the two *ortho* methyl carbon atoms. The ^1^H chemical shifts for the CH_2_, CH and OH protons of the ketone fragments in 1–6 show singlets at ∼5, ∼6.22 and ∼15.12 ppm respectively.

The ^125^Te NMR of all the isolated diorganotellurium(iv) derivatives (1–6) show the presence of only one tellurium containing species in solution, as well as in the solid state. A single resonance signal suggests they are stabile in solution state. The ^125^Te chemical shifts for 5 and 6 in CDCl_3_ move 78 ppm upfield from Cl to Br as expected in terms of increasing shielding of the tellurium atom.

## 3 Crystal structure of carbonyl functionalized organotellurium(iv) derivatives 1, 4 and 5

**Fig. 1:**
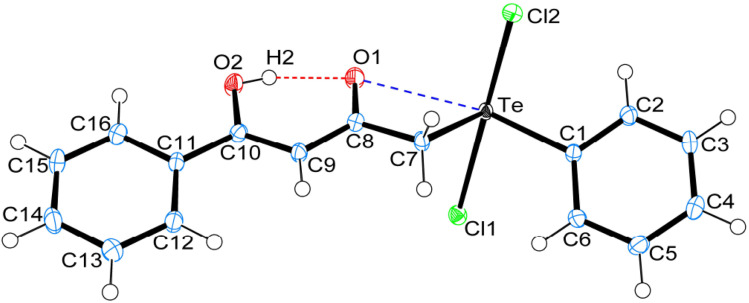
ORTEPs diagram showing 50% probability displacement ellipsoids and crystallographic numbering scheme for 1. Selected bond distances (Å) and angles (°): Te–C1 2.126(2), Te–C7 2.137(2), Te–Cl1 2.555(1), Te–Cl2 2.463(1), C8–O1 1.258(2), C10–O2 1.328(2), Te⋯O1, 2.861(1), O1⋯H2, 1.824(28), O2–H2, 0.783(29), O1⋯O2 2.559(2); C1–Te–C7 100.77(6), Te–C7–C8 104.66(9), Cl1–Te–Cl2 171.80(2), Te⋯O1⋯H2 165.45(85), O1⋯H2–O2 153.56(27), C7–Te⋯O1 54.34(4), C1–Te⋯O1 155.05(5), C10–O2⋯H2 104.46(2).

**Fig. 2:**
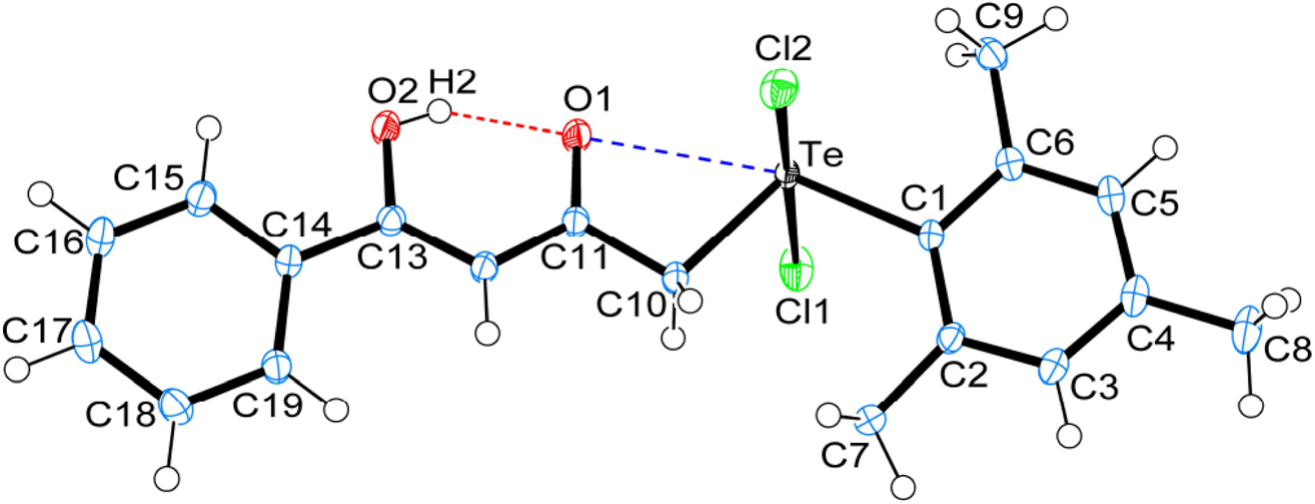
ORTEPs diagram showing 50% probability displacement ellipsoids and crystallographic numbering scheme for 4. Selected bond distances (Å) and angles (°): Te–C1 2.125(1), Te–C10 2.148(1), Te–Cl1 2.521(1), Te–Cl2 2.498(1), C11–O1 1.254(2), C13–O2 1.335(2), Te⋯O1, 2.847(1), O1⋯H2, 1.854(25), O2–H2, 0.756(25), O1⋯O2 2.541(2); C1–Te–C10 106.89(54), Te–C10–C11 105.37(9), Cl1–Te–Cl2 173.71(8), Te⋯O1⋯H2 174.65(78), O1⋯H2–O2 159.92(26), C10–Te⋯O1 54.62(4), C1–Te⋯O1 158.51(5), C13–O2⋯H2 107.92(2).

**Fig. 3:**
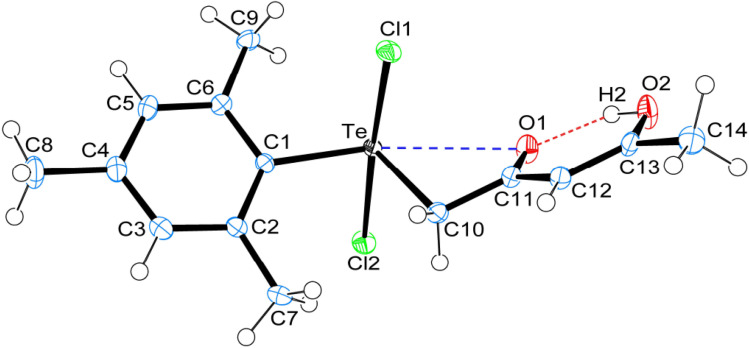
ORTEPs diagram showing 50% probability displacement ellipsoids and crystallographic numbering scheme for 5. Selected bond distances (Å) and angles (°): Te–C1 2.127(3), Te–C10 2.141(3), Te–Cl1 2.534(2), Te–Cl2 2.479(2), C11–O1 1.262(3), C13–O2 1.321(3), Te⋯O1, 2.926(3), O1⋯H2, 1.744(3), O2–H2, 0.840(3), O1⋯O2 2.498(4); C1–Te–C10 105.24(10), Te–C10–C11 104.29(16), Cl1–Te–Cl2 173.66(3), Te⋯O1⋯H2 153.18(12), O1⋯H2–O2 148.42(19), C10–Te⋯O1 52.97(8), C1–Te⋯O1 156.82(8), C13–O2⋯H2 109.44(2).

Crystal data and structure refinement details for compounds 1, 4 and 5 are given in Table S1.† ORTEP_S_ diagrams of 1, 4 and 5 are shown in Fig. 1, 2 and 3 respectively, each captioned with the selected interatomic distances and angles. Table S1† and packing diagrams of 1, 4 and 5 are presented in the ESI (Fig. S1–S10).† Compounds 1 and 4 crystallize in a monoclinic crystal system with the *P*2_1_/*c* space group, while 5 crystallizes in a monoclinic system with the *P*2_1_ space group. The primary geometry around the Te(iv) atom in these diorganotellurium dichlorides is ψ-trigonal bipyramidal with one equatorial position occupied by a stereochemically active lone pair. Interatomic Te⋯O(carbonyl) distances (*d*(Te⋯O) 2.861(1) Å in 1, 2.847(1) in 4 and 2.926(3) in 5) are longer than the Σ*r*_cov_ (Te,O) of 2.03 Å, and significantly shorter than Σ*r*_vdw_ (Te,O) of 3.58 Å, enough to imply the presence of attractive intramolecular 1,4-Te⋯O secondary bonding interactions.^25^ Moreover, the smaller Te–C–C(carbonyl) angle (104.66(9)° in 1, 105.37(9)° in 4 and 104.29(16)° in 5) compared to the tetrahedral angle indicate appreciable bending, consequentially Te(iv) and O atoms are attracted closer to each other. There are possible rotations about the Te–CH_2_ bonds, with the acetylacetone/benzoylacetone fragments in each case being oriented so that the carbonyl oxygen atoms are almost in the equatorial C–Te–C plane. Along with planarity, adjacent linearity of the C(*trans*)–Te⋯O triad(s) (155.05(5)° 1, 158.51(5)° in 4 and 156.82(8)° in 5) make n → σ* orbital interactions feasible. The observed intramolecular hydrogen bonding interaction in compound 5 (1.744(3) Å) is shorter than compounds 1 (1.824(28) Å) and 4 (1.854(25) Å), probably due to presence of an electron-donating methyl group on the hydroxyl carbon atom. The outstanding feature in all cases is a very short intramolecular O2–H⋯O1 hydrogen bond [*d*(O⋯O) in the range 2.498(4)–2.559(2) Å]. The downfield shifts of the *δ*(O–H) and *δ*(C–H) NMR signals are spectroscopic evidences of such strength. In the crystal structures the OH proton is observed to occupy a slightly asymmetric position, supporting all known solid state and solution spectroscopic data.^26^ Due to the presence of short strong intramolecular hydrogen bonding interactions, C=O and C=C bonds in the adopted six membered rings, together with the carbonyl oxygen and hydroxyl atoms, almost lie in a plane.

### 3.1 Supramolecular architectures in the crystal lattices of compound 1, 4 and 5

In addition to the short strong intramolecular hydrogen bonding interactions (O⋯H) and Te⋯O secondary bonding interactions, the other intermolecular hydrogen bonding interactions (C–H⋯O, C–H⋯Cl) and secondary bonding interactions Te⋯Cl and π⋯π that have been recognized to play a vital role in the self-assembly of carbonyl functionalized organotellurium(iv) dichlorides. Parametric details of such interactions are depicted in Table S2.†

The structure of 1 consists of a 2D helical structure running along the *b*-axis (Fig. S1†), with C–H⋯Cl interactions along the *c*-axis. Each Te(iv) atom is covalently bonded with two axial Cl atoms and two equatorial C-atoms. Simultaneously each Te(iv) atom is also interconnected with two SBIs [Te⋯O 2.861(1) Å and Te⋯Cl 3.477(1) Å] (Fig. S2†). The phenyl ring of benzylacetone fragment within each helical structure in 1 is glide such that the *para*-carbon atom of one molecule forms a π⋯π stacking interaction with the phenyl ring of benzylacetone fragment of a neighbouring molecule. The *para*-carbon⋯centroid distance between the phenyl ring of a neighbouring molecule in the structure of 1 is 3.652(2) Å (Fig. S3†). The crystal packing of 1 also consists of a centrosymmetric dimeric unit through C–H⋯Cl [2.861(1) Å] interactions repetition of these units gives rise to three-dimensional supramolecular architectures *via* self-assembly (Fig. S4†).^27,28^ The crystal structure of 4 consist of a centrosymmetric dimeric unit through reciprocal O–H⋯O [2.419(24) Å] intermolecular hydrogen bonding interaction (Fig. S5†). These dimeric units further connected with another dimeric unit through reciprocal C–H⋯Cl [2.902(0) Å] intermolecular hydrogen bonding interaction give rise to a 2D supramolecular architecture along *c*-axis (Fig. S6†). In the dimeric unit of 4 also consist of two strong intramolecular hydrogen bonding interaction C9–H9B⋯Cl2 [2.881(0) Å] and C7–H7B⋯Cl1 [2.956(0) Å]. Probably due to these interactions and steric effect of mesityl ring in compound 4 exhibits separate signals for both *ortho* methyl proton (2.74 & 2.79 ppm) and for both *meta* proton (6.43 & 6.97 ppm).

The crystal structure of 5 is devoid of Te based intermolecular SBIs. Steric demand of the mesityl group, acetylacetone fragment and small inter-atomic distance between Te and the axial Cl ligands make the Te atom inaccessible for intermolecular bonding. As a result, chlorine atoms are interconnected through the bifurcated and trifurcated C–H⋯Cl interactions, along with trifurcated C–H⋯O inter- and intra-connected hydrogen bonding interaction, giving rise to three-dimensional supramolecular packing (Fig. S7–S9†). Among all three Te(iv) dichlorides, the observed (O–H⋯O, ∼1.95 Å; C–H⋯O, ∼2.47 Å; C–H⋯Cl, ∼2.80 Å and Te⋯Cl, 3.45 Å) hydrogen bonding and SBIs are longer than Σ*r*_cov_ [(H,O), 0.97 Å; (H,Cl), 1.33 Å; (Te,Cl), 2.40 Å] and significantly shorter than [Σ*r*_vdw_ (H,O), 2.48 Å; (H,Cl), 2.81 Å; (Te,Cl), 3.83 Å] respectively.^25^ Linearity of the C–H⋯O and C–H⋯Cl make n → σ* orbital interaction possible. The four electron-three centre covalent bonding interaction and SBIs play a major role in the formation of supramolecular synthons.

## 4 Experimental

### 4.1. General

Preparative work was performed under dry nitrogen. Phenyl, *p*-tolyl, 1-naphthyl, and mesityl tellurium trichlorides were prepared by chlorination of their corresponding ditellurides. Commercial acetylacetone and benzoylacetone were dried with anhydrous Na_2_SO_4_ and freshly distilled under inert atmosphere. Melting points were recorded in capillary tubes and are uncorrected. The ^1^H, ^13^C{^1^H}, and ^125^Te NMR spectra were recorded on Bruker AMX 400, JEOL 400 or JEOL 300 spectrometers. Chemical shifts cited were referenced to TMS (^1^H, ^13^C{^1^H}) as internal and Me_2_Te (^125^Te) as external standard. Microanalyses were carried out using a Carlo Erba 1108 elemental analyzer. Tellurium was estimated volumetrically.

### 4.2. Syntheses

#### 4.2.1 Reactions of acetylacetone/benzoylacetone with ArTeCl_3_ (Ar = Ph, *p*-tolyl, 1-napthyl, and mesityl)

(1) A mixture of phenyltellurium trichloride (0.62 g, 2.0 mmol) and benzoylacetone (0.34 g, 2.1 mmol) was stirred slowly at ∼60 °C under a flow of dry nitrogen (∼10 h). The resulting paste was washed with cold petroleum ether (3 × 10 mL), triturated with diethyl ether and filtered to remove excess ketone. The residue was dissolved in dichloromethane (25 mL) and filtered through a short silica column. Concentration of the extract to about one third of its original volume and addition of diethyl ether (10 mL) afforded a pale orange solid which was recrystallized from benzene to give 1 as colourless needle shaped crystals. Yield: (0.18 g, 21%); mp 127 °C (from C_6_H_6_). (Found: C, 44.1; H, 3.3; Te, 29.2. C_16_H_14_Cl_2_O_2_Te requires C, 44.0; H, 3.2; Te, 29.2%); *δ*_H_ (300.1 MHz; CDCl_3_; Me_4_Si) 4.78 (2H, s, CH_2_), 6.34 (1H, s, CH), 7.44–7.50 (2H, m, aryl), 7.55–7.61 (4H, m, aryl), 7.87–7.89 (2H, m, aryl), 8.21–8.27 (2H, m, aryl), 15.31 (1H, br, OH); *δ*_C_ (100.5 MHz; CDCl_3_; Me_4_Si) 62.26 (CH_2_), 96.53 (CH), 127.26, 128.80, 130.05, 131.85, 133.05, 133.19, 133.71 (C–Ph), 181.67 (COH), 188.02 (CO); *δ*_Te_ (126.2 MHz; CDCl_3_; Me_2_Te) 860.3 (s).

(2) Prepared from *p*-tolyltellurium trichloride (0.65 g, 2.0 mmol) and benzoylacetone (0.34 g, 2.1 mmol) in a way similar to 1. Yield (0.34 g, 38%); mp 128 °C (from C_6_H_6_). (Found: C, 45.2; H, 3.6; Te, 28.1. C_17_H_16_Cl_2_O_2_Te requires C, 45.3; H, 3.6; Te, 28.3%); *δ*_H_ (300.1 MHz; CDCl_3_; Me_4_Si) 2.44 (3H, s, Me), 4.76 (2H, s, CH_2_), 6.34 (1H, s, CH), 7.38 (2H, d *J* 2.7 Hz, aryl), 7.41–7.50 (2H, m, aryl), 7.55–7.60 (1H, m, aryl), 7.87–7.91 (2H, m, aryl), 8.08–8.14 (2H, m, aryl), 15.31 (1H, br, OH); *δ*_C_ (100.5 MHz; CDCl_3_; Me_4_Si) 21.34 (Me), 61.85 (CH_2_), 96.61 (CH), 126.42, 127.24, 128.77, 130.78, 133.11, 133.14, 133.53, 142.66 (C–Ph), 181.70 (COH), 187.96 (CO); *δ*_Te_ (126.2 MHz; CDCl_3_; Me_2_Te) 863.8 (s).

(3) Prepared from 1-naphthyltellurium trichloride (0.72 g, 2.0 mmol) and benzoylacetone (0.34 g, 2.1 mmol) in a way similar to 1. Yield (0.81 g, 83%); mp 145 °C (from C_6_H_6_). (Found: C, 49.3; H, 3.4; Te, 26.1. C_20_H_16_Cl_2_O_2_Te requires C, 49.3; H, 3.3; Te, 26.2%); *δ*_H_ (300.1 MHz; CDCl_3_; Me_4_Si) 5.10 (2H, s, CH_2_), 6.46 (1H, s, CH), 7.49 (2H, t, *J* 1.9 Hz, aryl), 7.57–7.62 (1H, m, aryl), 7.66 (2H, t, *J* 1.9 Hz, aryl), 7.73 (1H, t, *J* 1.9 Hz, aryl), 7.93 (2H, d, *J* 2.0 Hz, aryl), 7.97 (1H, d, *J* 2.0 Hz, aryl), 8.08 (1H, d, *J* 2.1 Hz, aryl), 8.15 (1H, d, *J* 2.1 Hz, aryl), 8.21 (1H, d, *J* 1.8 Hz, aryl), 15.32 (1H, br, OH); *δ*_C_ (100.5 MHz; CDCl_3_; Me_4_Si) 62.57 (CH_2_), 96.23 (CH), 126.37, 126.70, 127.25, 128.17, 128.79, 130.70, 132.22, 132.69, 132.77, 133.23, 133.93, 134.22 (C-aryl), 181.05 (COH), 189.08 (CO); *δ*_Te_ (126.2 MHz; CDCl_3_; Me_2_Te) 779.5 (s).

(4) Prepared from mesityltellurium trichloride (0.72 g, 2.0 mmol) and benzoylacetone (0.34 g, 2.1 mmol) in a way similar to 1. Yield (0.33 g, 35%); mp 148 °C (from C_6_H_6_). (Found: C, 47.7; H, 4.3; Te, 26.7. C_19_H_20_Cl_2_O_2_Te requires C, 47.7; H, 4.2; Te, 26.6%); *δ*_H_ (300.1 MHz; CDCl_3_; Me_4_Si) 2.32 (3H, s, *p*-Me), 2.74 (3H, s, *o*-Me), 2.79 (3H, s, *o*-Me), 5.09 (2H, s, CH_2_), 6.43 (1H, s, CH), 6.97 (1H, s, aryl), 6.99 (1H, s, aryl), 7.48 (2H, t, *J* 1.9 Hz, aryl), 7.58 (1H, t, *J* 1.9 Hz, aryl), 7.91 (2H, d, *J* 1.9 Hz, aryl), 15.34 (1H, br, OH); *δ*_C_ (100.5 MHz; CDCl_3_; Me_4_Si) 21.01 (*p*-Me), 23.64 (Me), 23.76 (Me), 60.46 (CH_2_), 96.48 (CH), 127.30, 128.83, 130.41, 131.59, 133.01, 133.22, 135.47, 139.79, 141.00, 142.34 (C-Mes), 181.38 (COH), 189.40 (CO); *δ*_Te_ (126.2 MHz; CDCl_3_; Me_2_Te) 802.9 (s).

(5) Prepared from mesityltellurium trichloride (0.72 g, 2.0 mmol) and acetylacetone (1.5 mL, 15 mmol) in a way similar to 1. Yield (0.40 g, 48%); mp 145 °C (from C_6_H_6_). (Found: C, 40.2; H, 4.6; Te, 30.5. C_14_H_18_Cl_2_O_2_Te requires C, 40.3; H, 4.4; Te, 30.6%); *δ*_H_ (300.1 MHz; CDCl_3_; Me_4_Si) 2.14 (3H, s, *p*-Me), 2.32 (3H, s, Me), 2.70 (3H, s, *o*-Me), 2.80 (3H, s, *o*-Me), 4.95 (2H, s, CH_2_), 5.76 (1H, s, CH), 6.99 (1H, s, aryl), 7.03 (1H, s, aryl), 14.7 (1H, br, OH); *δ*_C_ (100.5 MHz; CDCl_3_; Me_4_Si) 21.00 (*p*-Me), 23.50 (*o*-Me), 23.60 (*o*-Me), 23.7 (Me), 60.10 (CH_2_), 100.10 (CH), 130.30, 131.50, 135.20, 139.80, 141.00, 142.30 (C-Mes), 188.10 (COH), 188.40 (CO); *δ*_Te_ (126.2 MHz; CDCl_3_; Me_2_Te) 801.0 (s).

#### 4.2.2 metathetical reactions of 5

Compound 6 was obtained in a good yield when 5 (0.42 g, 1.0 mmol) and NaBr (0.21 g, 2.0 mmol) were stirred together in chloroform (15 mL) for ∼3 h. Sodium bromide was removed by filtration. Addition of petroleum ether (5 mL) and cooling to 0 °C afforded yellow crystals of 6. Yield (0.48 g, 95%); mp 120 °C (from C_6_H_6_). (Found: C, 33.3; H, 3.6; Te, 25.3. C_14_H_18_Br_2_O_2_Te requires C, 33.2; H, 3.6; Te, 25.2%); *δ*_H_ (300.1 MHz; CDCl_3_; Me_4_Si) 2.14 (3H, s, Me), 2.32 (3H, s, *p*-Me), 2.66 (3H, s, *o*-Me), 2.76 (3H, s, *o*-Me), 5.10 (2H, s, CH_2_), 5.76 (1H, s, CH), 6.96 (1H, s, aryl), 7.02 (1H, s, aryl), 14.7 (1H, br, OH); *δ*_C_ (100.5 MHz; CDCl_3_; Me_4_Si) 20.97 (*p*-Me), 23.36 (*o*-Me), 23.40 (*o*-Me), 24.24 (Me), 59.02 (CH_2_), 99.92 (CH), 130.45, 131.58, 131.87, 139.39, 141.12, 142.27 (C-Mes), 187.69 (COH), 188.83 (CO); *δ*_Te_ (126.2 MHz; CDCl_3_; Me_2_Te) 722.8 (s).

#### 4.2.3 Attempted reductions of 1–5

Individual solutions of 1–5 (1.0 mmol) in dichloromethane (∼50 mL) were shaken with an aqueous solution of Na_2_S_2_O_5_ (0.19 g, 1.0 mmol) for 10 min. The organic layer gradually turned yellow it was separated and washed with water (4 × 50 mL). The organic fraction was dried over anhydrous Na_2_SO_4_ and filtered. Volatiles were removed under reduced pressure to give the respective diarylditellurides, Ar_2_Te_2_ as orange coloured solids, instead of the expected alkylaryltellurides.

### 4.3. Crystallography

Single crystals of 1, 4 and 5 suitable for X-ray crystallography were grown by slow evaporation of their C_6_H_6_ solutions at room temperature. ORTEPs and packing diagrams were generated with Ortep 3 for windows^29^ and DIAMOND 3.2 program respectively.^30^ Intensity data were collected on a Bruker SMART Apex CCD diffractometer at 100(2) K with graphite-monochromated Mo-Kα (0.7107 Å) radiation. The data was integrated with SAINT software.^31^ An experimental absorption modification was applied to the collected reflections with SADABS.^32^ The structure was confirmed by direct methods using SHELXTL and was refined on F2 by the full-matrix least-squares procedure using the program SHELXL-2018.^33^ All non-hydrogen atoms were refined with anisotropic displacement parameters. Hydrogen atoms attached to carbon were included in geometrically calculated positions using a riding model and were refined isotropically. Packing diagrams for the molecular structures of 1, 4 and 5 are shown in Fig. S1–S10 of the ESI.† Additionally, the ^1^H, ^13^C{^1^H} and ^125^Te NMR spectra are also included in the ESI (Fig. S11–S38).†

## 5 Conclusions

Electrophilic substitution reaction of aryltellurium trichlorides, ArTeCl_3_ (Ar = C_6_H_5_, Ph; 4-Me-C_6_H_4_, *p*-Tol; 1-C_10_H_7_, 1-Nap; 2,4,6-Me_3_C_6_H_2_, Mes) with acetylacetone/benzoylacetone to give carbonyl functionalized unsymmetrical diorganotellurium(iv) dichlorides, Ph[PhC(OH)CHC(O)CH_2_]TeCl_2_ (1), *p*-Tol[PhC(OH)CHC(O)CH_2_]TeCl_2_ (2), 1-Nap[PhC(OH)CHC(O)CH_2_]TeCl_2_ (3), Mes[PhC(OH)CHC(O)CH_2_]TeCl_2_ (4) and Mes[MeC(OH)CHC(O)CH_2_]TeCl_2_ (5). Mes[PhC(OH)CHC(O)CH_2_]TeBr_2_ (6) can also be obtained with 95% yield from the corresponding dichlorides by metathesis with NaBr in chloroform at room temperature. Biphasic (H_2_O/CH_2_Cl_2_) reduction of all the dichlorides with Na_2_S_2_O_5_ affords labile tellurides, which decompose quickly even at room temperature into the more stable symmetric ditellurides, Ar_2_Te_2_. Mesityl fragments bearing organotellurium(iv) derivatives show separate ^1^H and ^13^C{^1^H} NMR signals for the *ortho* methyls and meta protons of the mesityl ring.

## Data availability

The data supporting this article have been included as part of the ESI.† Crystallographic data for compounds 1, 4 & 5 has been deposited at the Cambridge Crystallographic Data Centre (CCDC) under accession numbers 2378222, 2378223 and 2378224 and can be obtained from “https://www.ccdc.cam.ac.uk/”.

## Conflicts of interest

There are no conflicts of interest to declare.

## Supplementary Material

RA-014-D4RA06023G-s001

RA-014-D4RA06023G-s002
