# Supplementary material for: Synthesis and characterization of carbonyl functionalized organotellurium(iv) derivatives
Source: RSC Adv. 2024 Nov 8;14(48):35650–6. doi: 10.1039/d4ra06023g (PMC11545911; doi:10.1039/d4ra06023g)
Supplement: RA-014-D4RA06023G-s001 [file RA-014-D4RA06023G-s001.pdf]

Supplementary Information (SI) for RSC Advances.
This journal is © The Royal Society of Chemistry 2024

# Supporting Info file

# for

## Synthesis and Characterization of Carbonyl Functionalized Organotellurium(IV) Derivatives

Puspendra Singh,*[a] Mariya Khan,[b] Andrew Duthie,[c] Ray J. Butcher[d]

[a]Department of Chemistry, Dr. Shakuntala Misra National Rehabilitation University, Lucknow, 226017, India.

[b]Department of Chemistry, University of Lucknow, Lucknow, 226007, India.

[c]Department of Chemistry, Indian Institute of Technology Kanpur, Kanpur, 208016, India

[d]Department of Chemistry, Howard University, Washington DC20059, USA

*Corresponding Author, E-mail address: pushpendrasingh0612@gmail.com

## INDEX


| S.No. | Content | Page No. |
|---|---|---|
| | **Index** | **S1-S3** |
| 1 | **Table S1**. Crystal Data and Structure Refinement Details of 1, 4 and 5 | **S4** |
| 2 | **Table S2**. Hydrogen bonds for **1**, **4** and **5** (Å and °). | **S5** |
| 3 | **Fig. S1** Crystal lattices of compound **1** showing helical structure through C-H---Cl (green) hydrogen bonding interactions. | **S6** |
| 4 | **Fig. S2** Crystal lattices of compound **1** showing O-H---O (red), C-H---Cl and (green) hydrogen bonding interactions and Te---O (blue) & Te---Cl (black) secondary bonding interaction. | **S7** |
| 5 | **Fig. S3** Crystal lattices of compound **1** showing π---π (brown) interaction. | **S7** |
| 6 | **Fig. S4.** Centrosymmetric dimeric unit in the crystal lattices of compound **1** through C-H---Cl (green) hydrogen bonding interactions. | **S8** |
| 7 | **Fig. S5.** Centrosymmetric dimeric unit in the crystal lattices of compound **4** through O-H---O (purple) hydrogen bonding interactions, Te---O (blue) secondary bonding interaction. | **S8** |
| 8 | **Fig. S6.** Supramolecular architecture along c axis of compound **4**. | **S9** |

| | | |
|---|---|---|
| 9 | **Fig. S7.** Supramolecular architecture along c axis of compound **4**. | **S9** |
| 10 | **Fig. S8-S10** Supramolecular architectures in the crystal lattices of compound **5**. | **S10-S11** |
| 11 | **Fig. S11** $^{1}H$ NMR spectrum of compound $Ph[PhC(OH)CHC(O)CH_2]TeCl_2$ (**1**) in $CDCl_3$. | **S12** |
| 12 | **Fig. S12** Expanded aryl region of $^{1}H$ NMR spectrum of compound $Ph[PhC(OH)CHC(O)CH_2]TeCl_2$ (**1**) in $CDCl_3$. | **S13** |
| 13 | **Fig. S13** $^{13}C$ NMR spectrum of compound $Ph[PhC(OH)CHC(O)CH_2]TeCl_2$ (**1**) in $CDCl_3$. | **S14** |
| 14 | **Fig. S14** Expanded aryl region of $^{13}C$ NMR spectrum of compound $Ph[PhC(OH)CHC(O)CH_2]TeCl_2$ (**1**) in $CDCl_3$. | **S15** |
| 15 | **Fig. S15** $^{125}Te$ NMR spectrum of compound $Ph[PhC(OH)CHC(O)CH_2]TeCl_2$ (**1**) in $CDCl_3$. | **S16** |
| 16 | **Fig. S16** $^{1}H$ NMR spectrum of compound *p*-$Tol[PhC(OH)CHC(O)CH_2]TeCl_2$ (**2**) in $CDCl_3$. | **S17** |
| 17 | **Fig. S17** Expanded aryl region of $^{1}H$ NMR spectrum of compound *p*-$Tol[PhC(OH)CHC(O)CH_2]TeCl_2$ (**2**) in $CDCl_3$. | **S18** |
| 18 | **Fig. S18** $^{13}C$ NMR spectrum of compound *p*-$Tol[PhC(OH)CHC(O)CH_2]TeCl_2$ (**2**) in $CDCl_3$. | **S19** |
| 19 | **Fig. S19** Expanded aryl region of $^{13}C$ NMR spectrum of compound *p*-$Tol[PhC(OH)CHC(O)CH_2]TeCl_2$ (**2**) in $CDCl_3$. | **S20** |
| 20 | **Fig. S20** $^{125}Te$ NMR spectrum of compound *p*-$Tol[PhC(OH)CHC(O)CH_2]TeCl_2$ (**2**) in $CDCl_3$. | **S21** |
| 21 | **Fig. S21** $^{1}H$ NMR spectrum of compound *1*-$Nap[PhC(OH)CHC(O)CH_2]TeCl_2$ (**3**) in $CDCl_3$. | **S22** |
| 22 | **Fig. S22** Expanded aryl region of $^{1}H$ NMR spectrum of compound *1*-$Nap[PhC(OH)CHC(O)CH_2]TeCl_2$ (**3**) in $CDCl_3$. | **S23** |
| 23 | **Fig. S23** $^{13}C$ NMR spectrum of compound *1*-$Nap[PhC(OH)CHC(O)CH_2]TeCl_2$ (**3**) in $CDCl_3$. | **S24** |
| 24 | **Fig. S24** Expanded aryl region of $^{13}C$ NMR spectrum of compound *1*-$Nap[PhC(OH)CHC(O)CH_2]TeCl_2$ (**3**) in $CDCl_3$. | **S25** |
| 25 | **Fig. S25** $^{125}Te$ NMR spectrum of compound *1*-$Nap[PhC(OH)CHC(O)CH_2]TeCl_2$ (**3**) in $CDCl_3$. | **S26** |
| 26 | **Fig. S26** $^{1}H$ NMR spectrum of compound $Mes[PhC(OH)CHC(O)CH_2]TeCl_2$ (**4**). | **S27** |
| 27 | **Fig. S27** Expanded aryl region of $^{1}H$ NMR spectrum of compound $Mes[PhC(OH)CHC(O)CH_2]TeCl_2$ (**4**). | **S28** |
| 28 | **Fig. S28** $^{13}C$ NMR spectrum of compound $Mes[PhC(OH)CHC(O)CH_2]TeCl_2$ (**4**) in $CDCl_3$. | **S29** |
| 29 | **Fig. S29** Expanded aryl region of $^{13}C$ NMR spectrum of compound $Mes[PhC(OH)CHC(O)CH_2]TeCl_2$ (**4**) in $CDCl_3$. | **S30** |
| 30 | **Fig. S30** $^{125}Te$ NMR spectrum of compound $Mes[PhC(OH)CHC(O)CH_2]TeCl_2$ (**4**) in $CDCl_3$. | **S31** |
| 31 | **Fig. S31** $^{1}H$ NMR spectrum of compound $Mes[CH_3(OH)CHC(O)CH_2]TeCl_2$ (**5**). | **S32** |
| 32 | **Fig. S32** Expanded aryl region of $^{1}H$ NMR spectrum of compound $Mes[CH_3(OH)CHC(O)CH_2]TeCl_2$ (**5**). | **S33** |
| 33 | **Fig. S33** $^{13}C$ NMR spectrum of compound $Mes[CH_3(OH)CHC(O)CH_2]TeCl_2$ (**5**) in $CDCl_3$. | **S34** |

| | | |
|---|---|---|
| 34 | **Fig. S34** Expanded alkyl region of $^{13}C$ NMR spectrum of compound Mes[$CH_3$(OH)CHC(O)$CH_2$]$TeCl_2$ (**5**) in $CDCl_3$. | **S35** |
| 35 | **Fig. S35** $^{1}H$ NMR spectrum of compound Mes[$CH_3$(OH)CHC(O)$CH_2$]$TeBr_2$ (**6**). | **S36** |
| 36 | **Fig. S36** Expanded aryl region of $^{1}H$ NMR spectrum of compound Mes[$CH_3$(OH)CHC(O)$CH_2$]$TeBr_2$ (**6**). | **S37** |
| 37 | **Fig. S37** Expanded aryl region of $^{13}C$ NMR spectrum of compound Mes[$CH_3$(OH)CHC(O)$CH_2$]$TeBr_2$ (**6**) in $CDCl_3$. | **S38** |
| 38 | **Fig. S38** $^{125}Te$ NMR spectrum of compound Mes[$CH_3$(OH)CHC(O)$CH_2$]$TeBr_2$ (**6**) in $CDCl_3$. | **S39** |

**Table S1. Crystal Data and Structure Refinement Details of 1, 4 and 5**

| | **1** | **4** | **5** |
|---|---|---|---|
| empirical formula | $C_{16}H_{14}Cl_2O_2Te$ | $C_{19}H_{20}Cl_2O_2Te$ | $C_{14}H_{18}Cl_2O_2Te$ |
| formula mass (g $mol^{-1}$) | 436.77 | 478.85 | 416.78 |
| Temp (K) | 100(2) | 123(2) | 100(2) |
| Wavelength, λ (Å) | 0.710 73 | 0.710 73 | 0.710 73 |
| cryst syst | monoclinic | monoclinic | monoclinic |
| cryst size ($mm^3$) | 0.55 x 0.10 x 0.09 | 0.77 x 0.55 x 0.35 | 0.44 x 0.27 x 0.23 |
| space group | $P2_1/c$ | $P2_1/c$ | $P2_1$ |
| *a* (Å) | 13.395(3) | 8.7845(4) | 8.389(5) |
| *b* (Å) | 13.251(3) | 19.5434(8) | 12.312(5) |
| *c* (Å) | 9.036(2) | 11.0751(5) | 8.461(5) |
| α (deg) | 90 | 90 | 90.000(5) |
| β (deg) | 98.213(4) | 97.679(4) | 116.320(5) |
| γ (deg) | 90 | 90 | 90.000(5) |
| Volume ($Å^3$) | 1587.5(7) | 1884.3(1) | 783.3(7) |
| Z | 4 | 4 | 2 |
| $\rho_{calcd}$ (Mg $m^3$) | 1.827 | 1.688 | 1.767 |
| abs coeff ($mm^{-1}$) | 2.211 | 1.871 | 2.235 |
| F(000) | 848 | 944 | 408 |
| $\theta$ range (deg) | 2.17-32.17 | 3.13-40.86 | 2.69-41.13 |
| index ranges | $-19 \leq h \leq 18$, $-18 \leq k \leq 19$, $-13 \leq l \leq 12$ | $-15 \leq h \leq 16$, $-35 \leq k \leq 27$, $-20 \leq l \leq 19$ | $-14 \leq h \leq 15$, $-22 \leq k \leq 22$, $-15 \leq l \leq 11$ |
| no. of rflns collected | 12748 | 24302 | 11130 |
| no. of indep rflns | 5046 (*R*(int) = 0.0344) | 11990 (*R*(int) = 0.0256) | 7370 (*R*(int) = 0.0295) |
| completeness to $\theta$max (%) | 99.4 | 99.8 | 99.5 |
| abs cor | semiempirical from equivalents | analytical | analytical |
| max. min. transmission | 0.7464, 0.4720 | 0.593, 0.434 | 0.733, 0.564 |
| refinement method | full-matrix least squares on $F^2$ | full-matrix least squares on $F^2$ | full-matrix least squares on $F^2$ |
| No. of data/restraints/ parameters | 5046/0/194 | 11990/0/224 | 7370/1/179 |
| goodness of fit on $F^2$ | 1.040 | 1.088 | 1.019 |
| final *R* indices [$I > 2\sigma(I)$) | R1 = 0.0224, wR2 = 0.0570 | R1 = 0.0345, wR2 = 0.0652 | R1 = 0.0292, wR2 = 0.0569 |
| *R* indices (all data) | R1 = 0.0255, wR2 = 0.0586 | R1 = 0.0515, wR2 = 0.0707 | R1 = 0.0344, wR2 = 0.0601 |
| largest diff peak/hole (e $Å^{-3}$) | 1.013/-0.549 | 0.687/-1.097 | 1.285/-0.985 |

**Table S2**. Hydrogen bonds for **1**, **4** and **5** (Å and °).

| | **D-H···A** | **d(D-H)** | **d(H···A)** | **d(D···A)** | **∠(DHA)** | **Symmetry** |
|---|---|---|---|---|---|---|
| **1** | O(2)-H(2)···O(1) | 0.80(3) | 1.824(3) | 2.559(2) | 154(3) | |
| | C(9)-H(9A)···Cl(1) | 0.95 | 2.667(1) | 3.541(2) | 153.1 | x,-y+1/2,z-1/2 |
| | C(6)-H(5A)···Cl(1) | 0.95 | 2.785(1) | 3.261(2) | 111.9 | -1+x, 0.5-y, -1.5+z |
| | C(9)-H(9A)···Cl(1) | 0.95 | 2.667(1) | 3.541(2) | 153.1 | 1-x, 0.5+y, 0.5-z |
| | C(15)-H(15A)···Cl(2) | 0.95 | 2.861(1) | 3.783(2) | 163.7 | -1-x, y, -1+z |
| | C(2)-H(2A)···Cl(2) | 0.95 | 2.853(1) | 3.302(2) | 110.1 | 1-x, 1-y, 1+z |
| | C(13)-H(13A)···O(1) | 0.95 | 2.427(1) | 3.339(2) | 160.9 | 1-x, 1-y, 1-z |
| **4** | O(2)-H(2)···O(1) | 0.75(2) | 1.854(2) | 2.541(1) | 151(3) | |
| | O(2)-H(2)···O(1) | 0.75(2) | 2.419(2) | 2.884(2) | 121(2) | -x+1,-y+1,-z+1 |
| | C(9)-H(9B)···Cl(2) | 0.98 | 2.881(0) | 3.629(2) | 133.8 | 1 -x, 1-y. 1-z |
| | C(7)-H(7B)···Cl(1) | 0.98 | 2.956(0) | 3.741(2) | 137.9 | 1 -x, 1-y. 1-z |
| | C(17)-H(17A)···Cl(1) | 0.95 | 2.902(0) | 3.717(2) | 144.6 | 1 -x, 1-y. 1-z |
| **5** | O(2)-H(2)···O(1) | 0.84 | 1.744(3) | 2.498(3) | 148.4 | |
| | C(12)-H(12)···Cl(2) | 0.95 | 2.911(1) | 3.704(3) | 141.8 | 1 -x+1,y-1/2,-z+1 |
| | C(7)-H(7B)···Cl(2) | 0.95 | 2.695(1) | 3.531(3) | 143.6 | 1-x, -0.5+y, 2-z |
| | C(14)-H(14A)···O(1) | 0.98 | 2.522(2) | 3.501(4) | 176.4 | -x+1,y-1/2,-z |
| | C(14)-H(14B)···Cl(1) | 0.98 | 2.843(1) | 3.803(4) | 166.8 | -x+2,y-1/2,-z+1 |
| | C(9)-H(9A)···Cl(1) | 0.98 | 2.872(1) | 3.749(3) | 149.6 | -x+2,y-1/2,-z+1 |
| | C(9)-H(9C)···Cl(1) | 0.98 | 2.778(1) | 3.462(3) | 127.5 | -x+2,y-1/2,-z+1 |

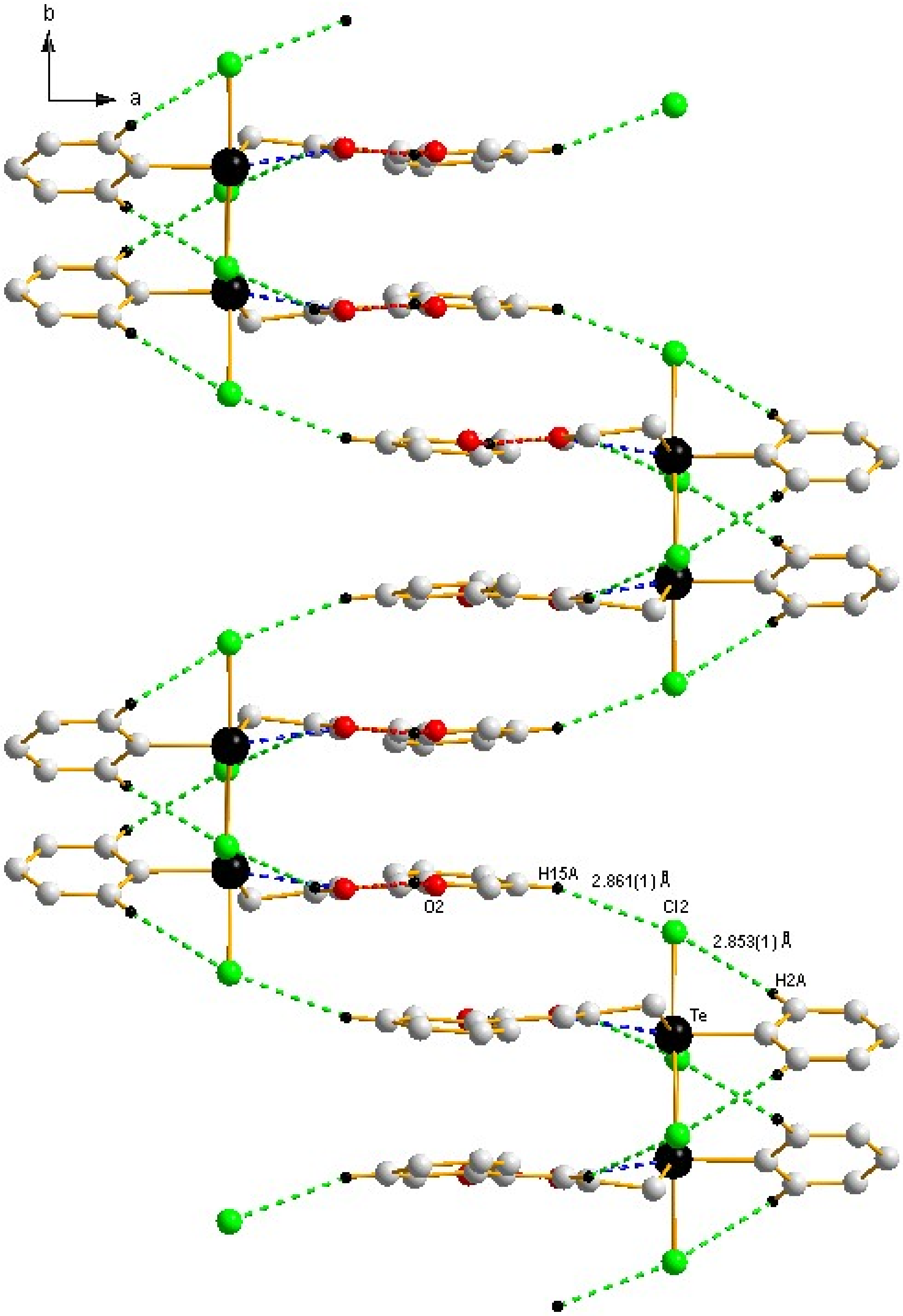


**Fig. S1** Crystal lattices of compound **1** showing helical structure through C-H---Cl (green) hydrogen bonding interactions.

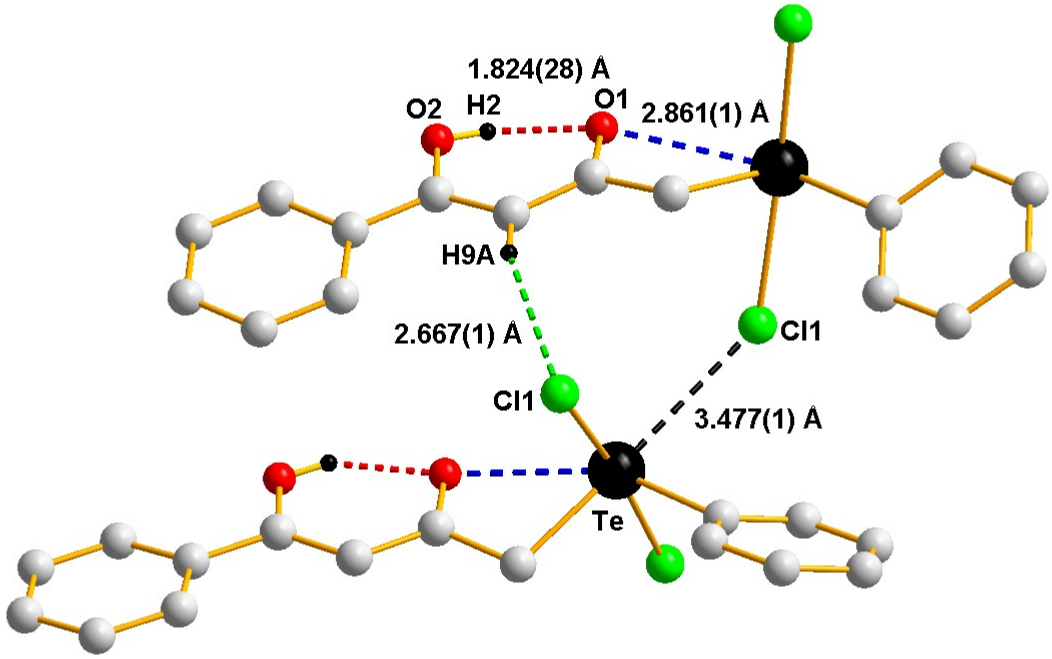


**Fig. S2** Crystal lattices of compound **1** showing O-H---O (red), C-H---Cl and (green) hydrogen bonding interactions and Te---O (blue) & Te---Cl (black) secondary bonding interaction.

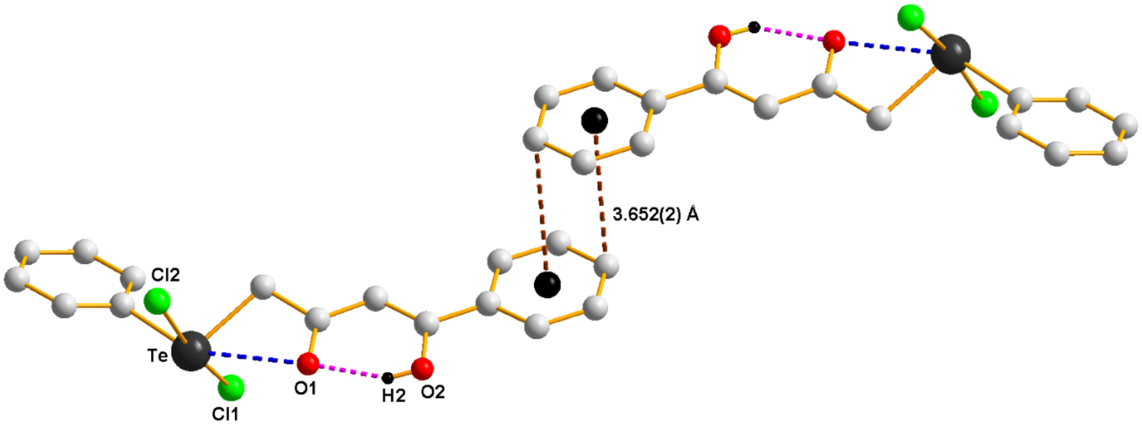


**Fig. S3** Crystal lattices of compound **1** showing π---π (brown) interaction.

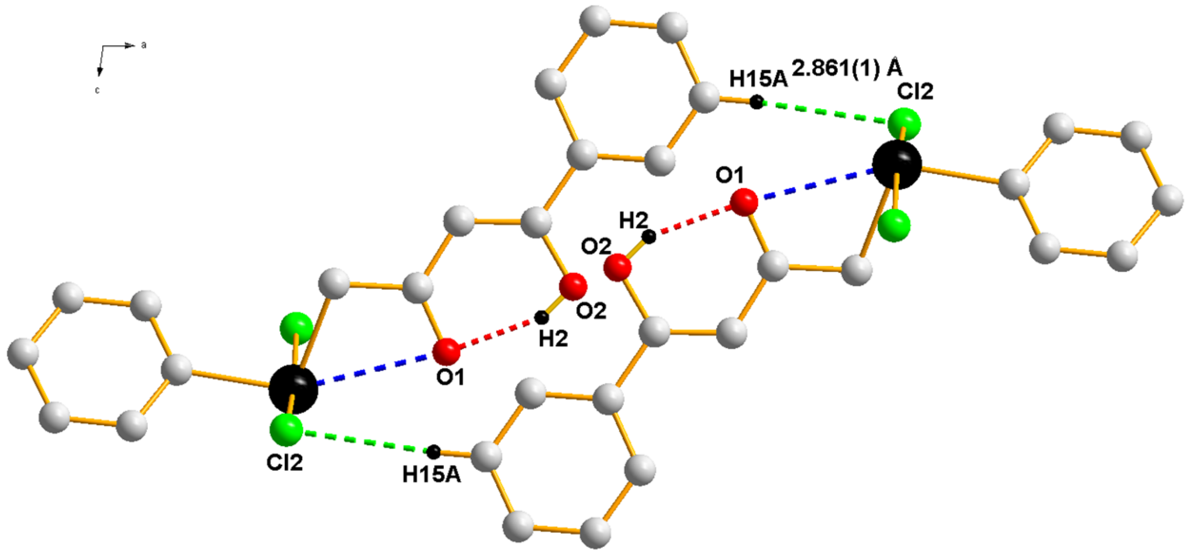


**Fig. S4.** Centrosymmetric dimeric unit in the crystal lattices of compound **1** through C-H---Cl (green) hydrogen bonding interactions.

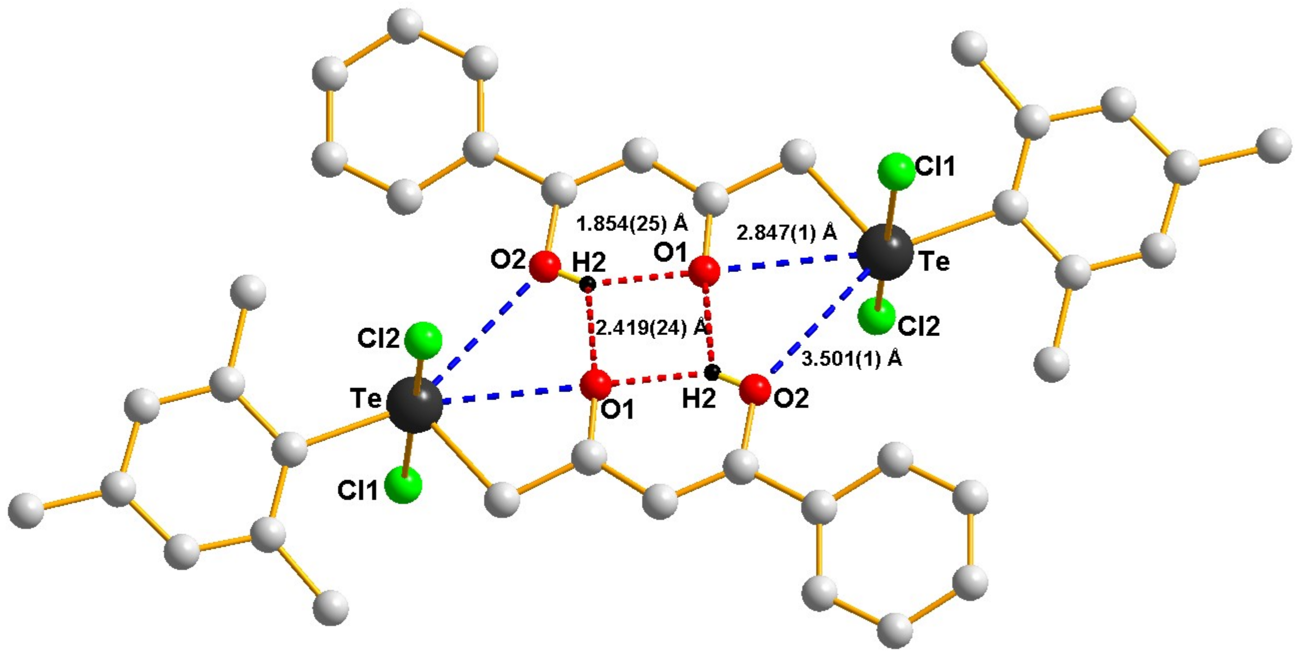


**Fig. S5.** Centrosymmetric dimeric unit in the crystal lattices of compound **4** through O-H---O (purple) hydrogen bonding interactions, Te---O (blue) secondary bonding interaction.

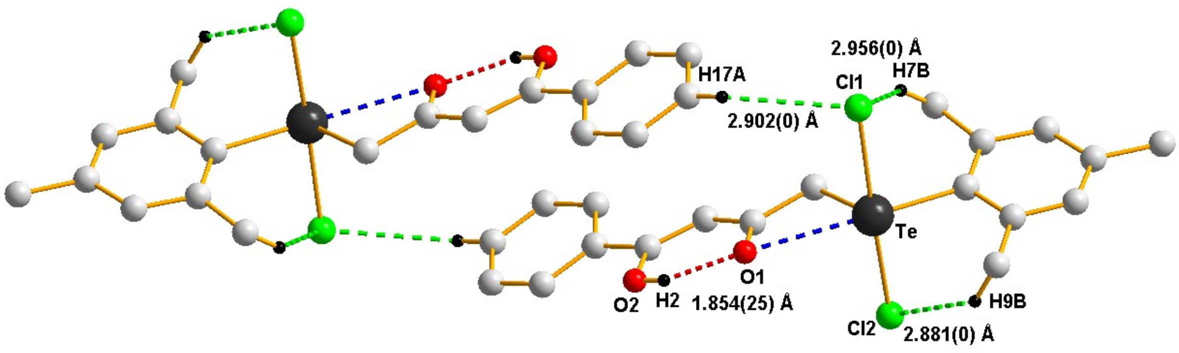


**Fig. S6.** Centrosymmetric dimeric unit in the crystal lattices of compound **4** through C-H---Cl (green) hydrogen bonding interactions.

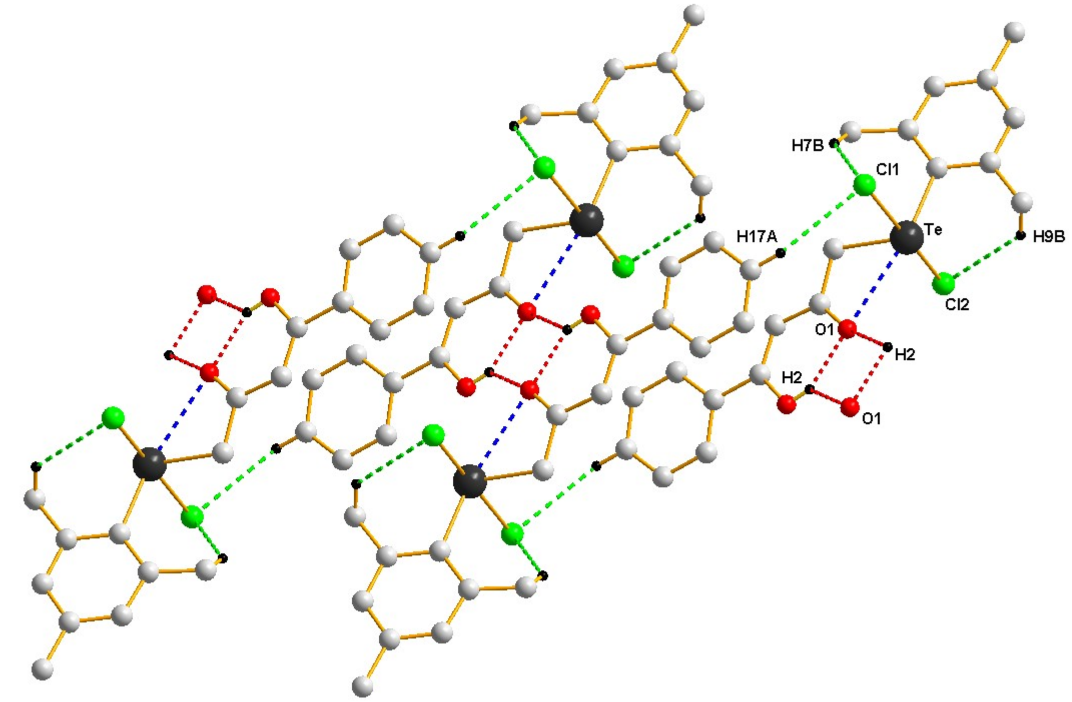


**Fig. S7.** Supramolecular architecture along c axis of compound **4**.

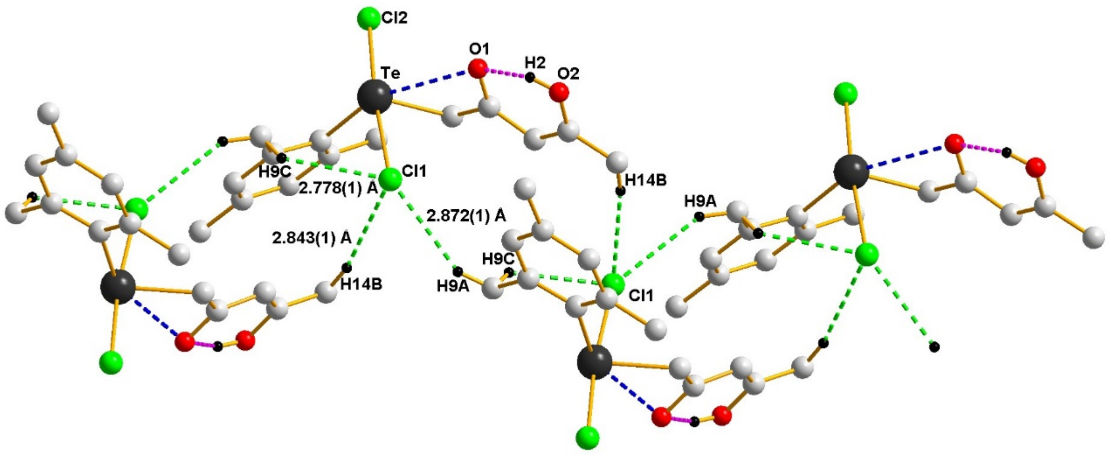


**Fig. S8.** Supramolecular architectures in the crystal lattices of compound **5** through O-H---O (purple) and C-H---Cl (green) hydrogen bonding interactions and Te---O (blue) secondary bonding interaction.

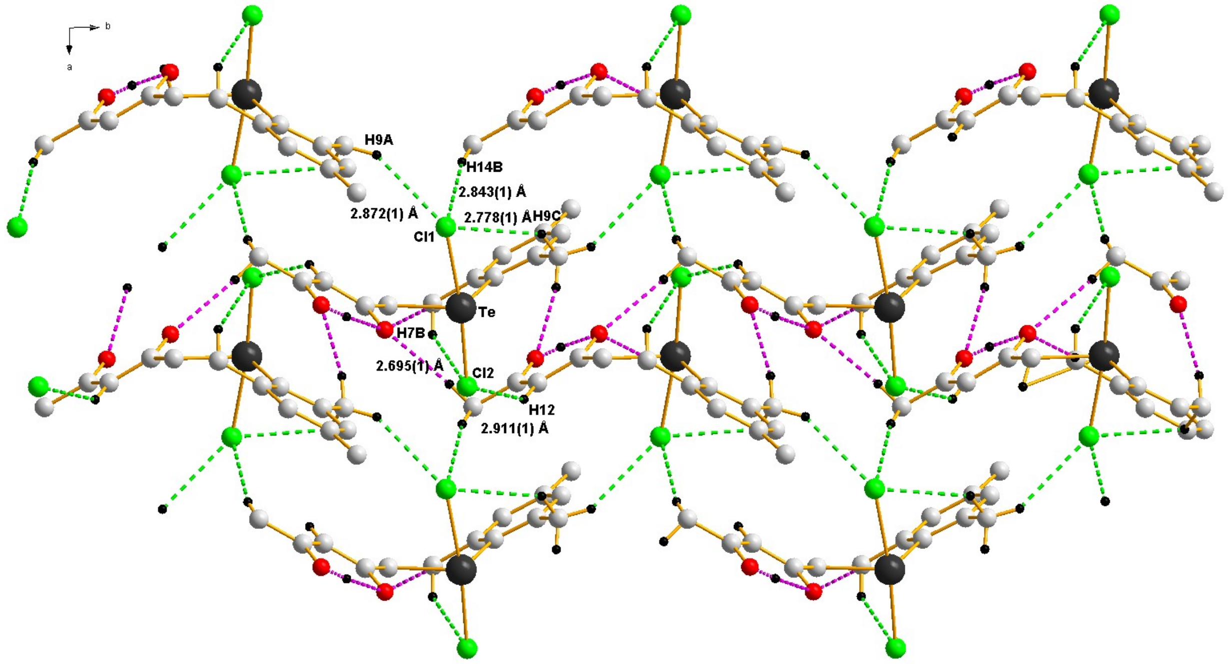


**Fig. S9.** Supramolecular architectures in the crystal lattices of compound **5** through O-H---O (purple) and C-H---Cl (green) hydrogen bonding interactions.

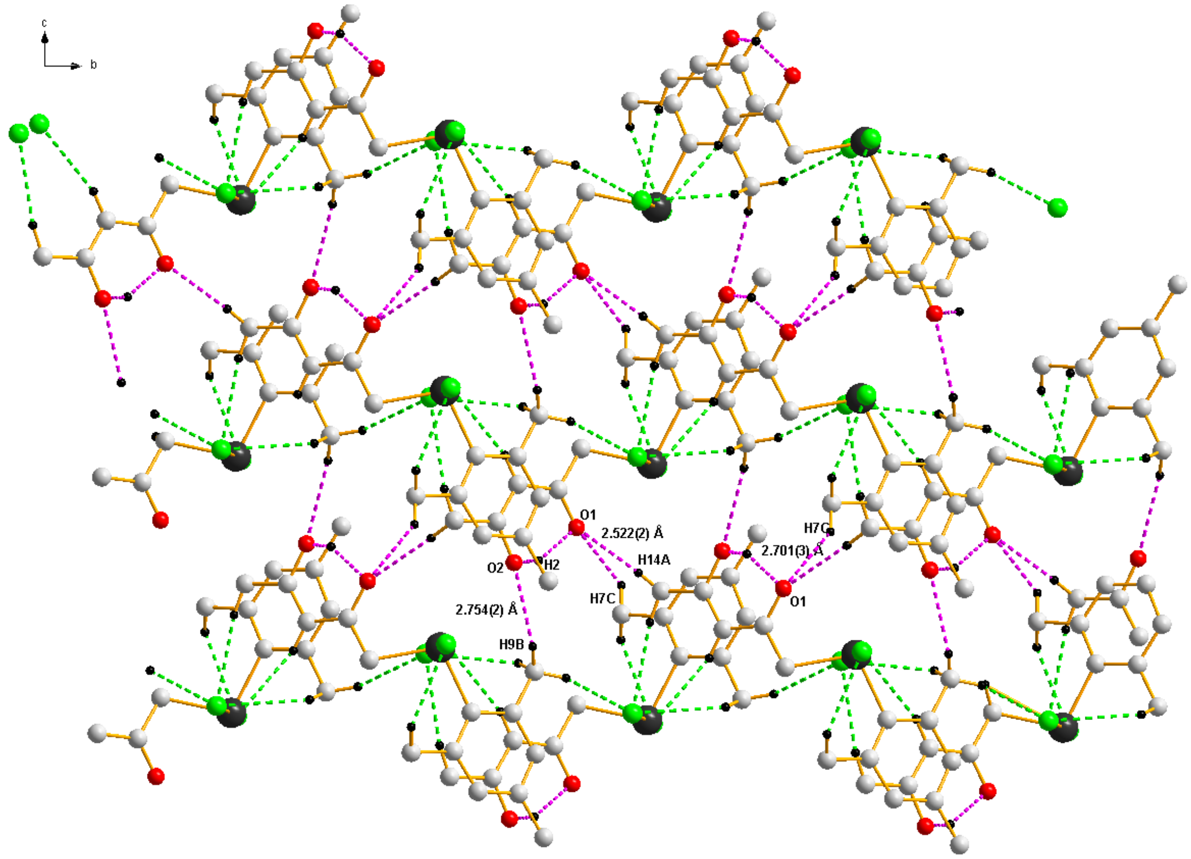


**Fig. S10.** Supramolecular architectures in the crystal lattices of compound **5** through O-H---O (purple) and C-H---Cl (green) hydrogen bonding interactions.

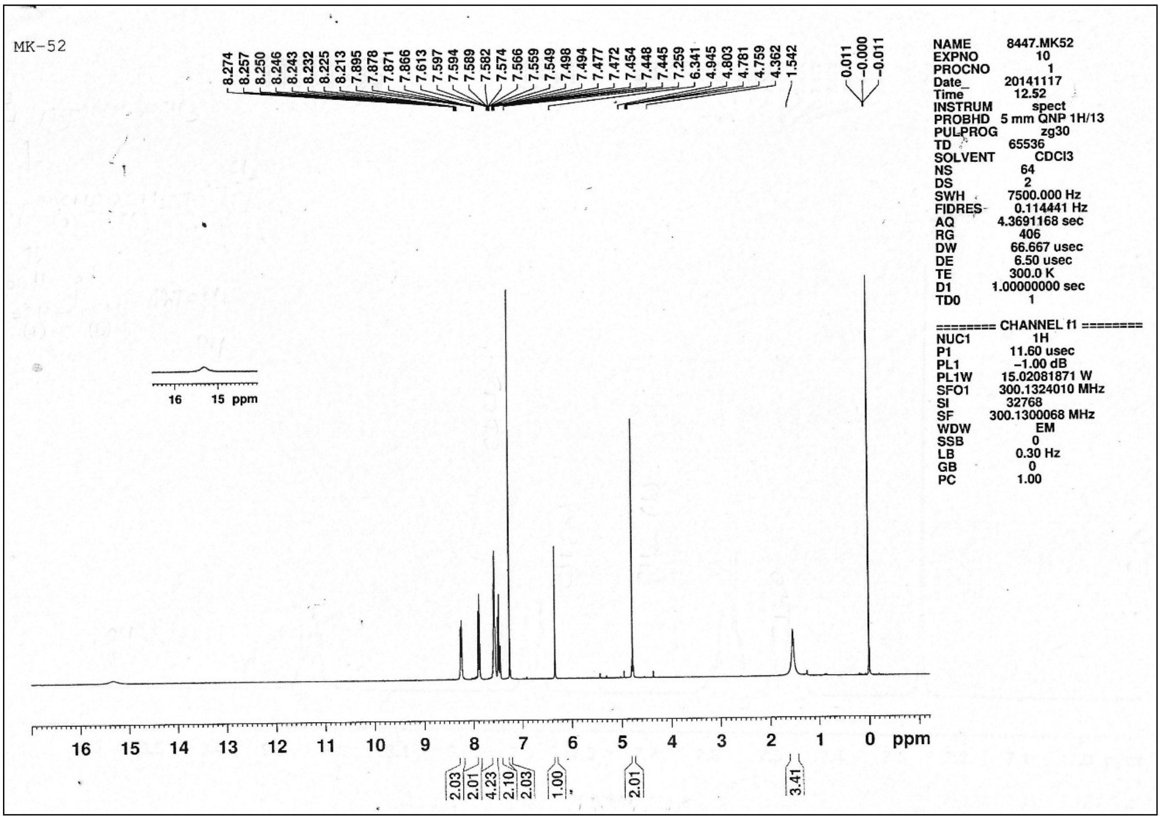


**Fig. S11.** $^{1}$H NMR spectrum of compound Ph[PhC(OH)CHC(O)$CH_2$]$TeCl_2$ (**1**) in $CDCl_3$.

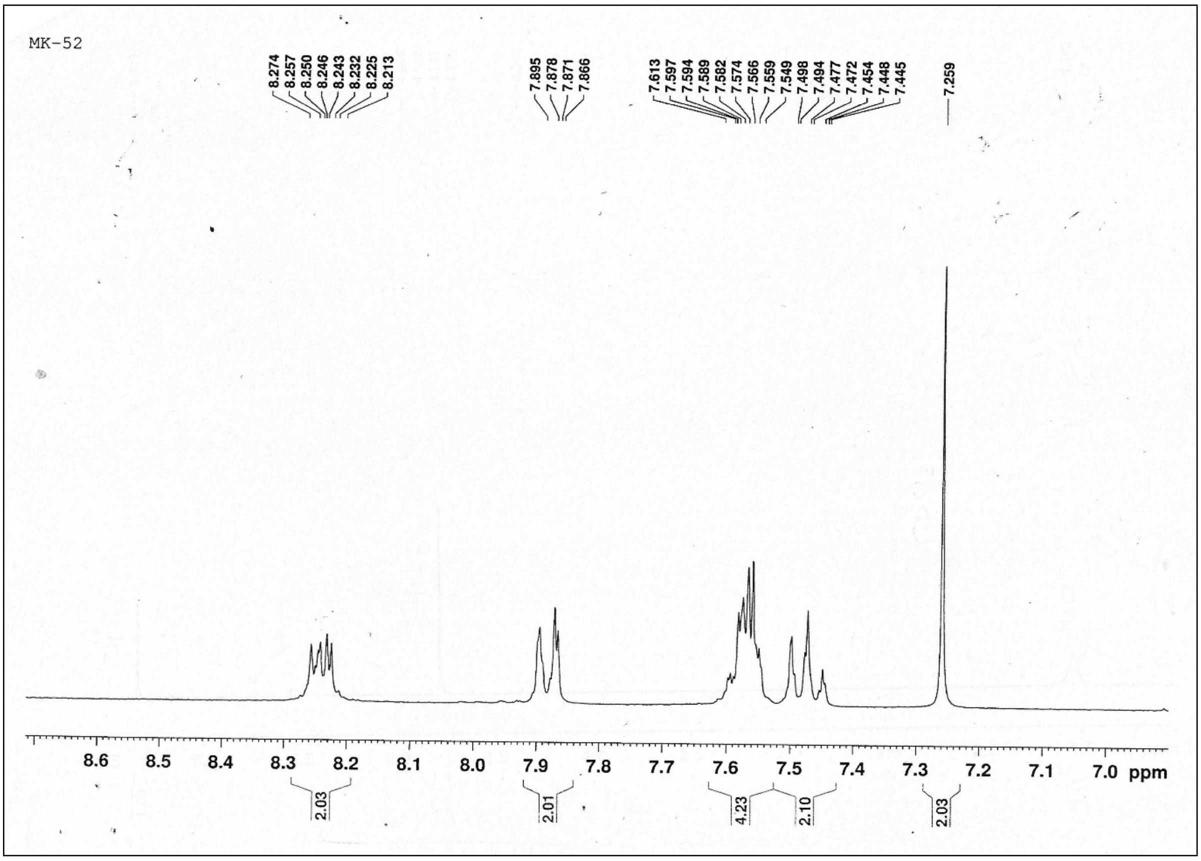


**Fig. S12.** Expanded aryl region of $^{1}$H NMR spectrum of compound Ph[PhC(OH)CHC(O)$CH_2$]$TeCl_2$ (**1**) in $CDCl_3$.

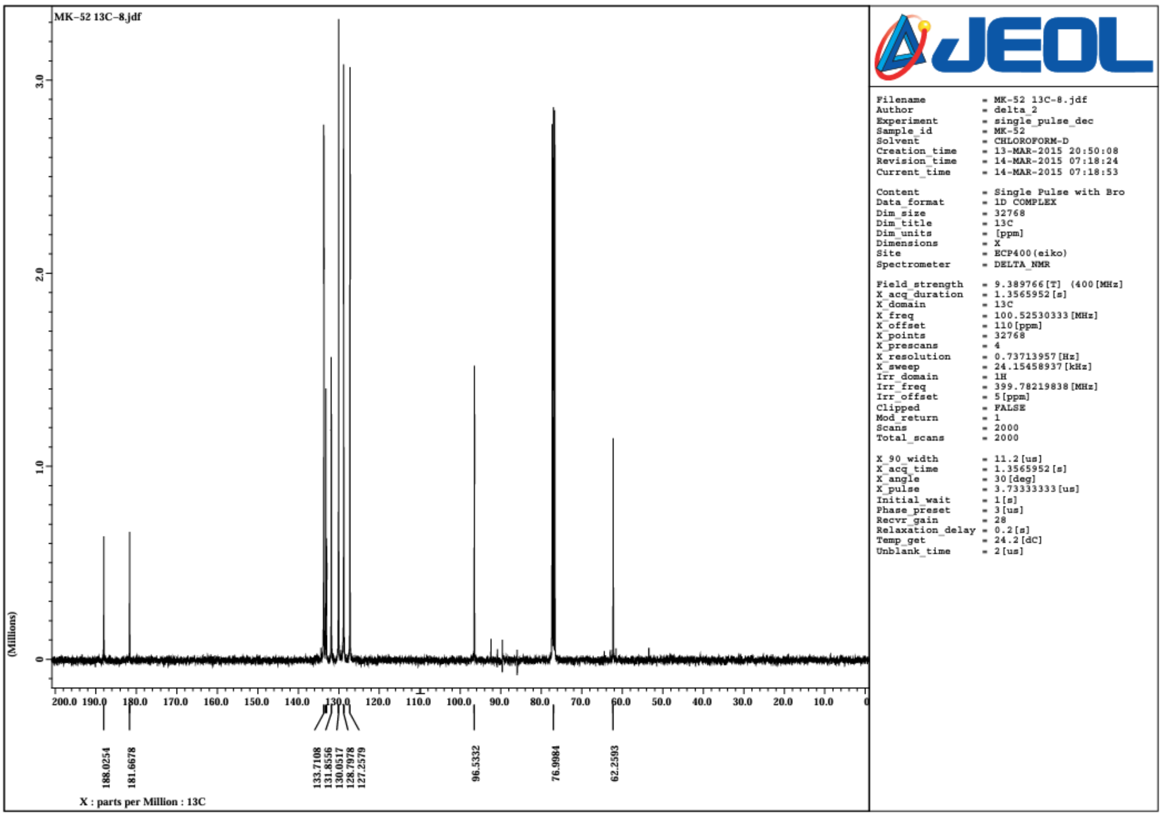


**Fig. S13.** $^{13}C$ NMR spectrum of compound Ph[PhC(OH)CHC(O)$CH_2$]$TeCl_2$ (**1**) in $CDCl_3$.

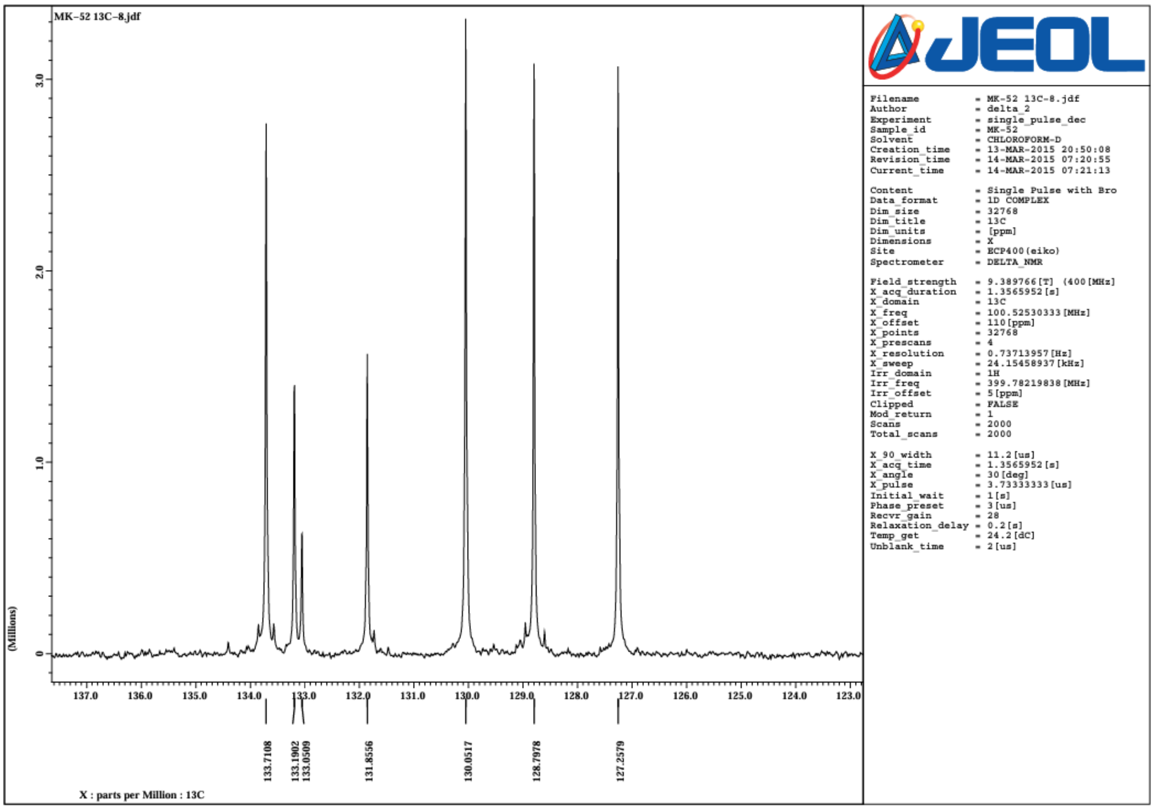


**Fig. S14.** Expanded aryl region of $^{13}$C NMR spectrum of compound Ph[PhC(OH)CHC(O)$CH_2$]$TeCl_2$ (**1**) in $CDCl_3$.

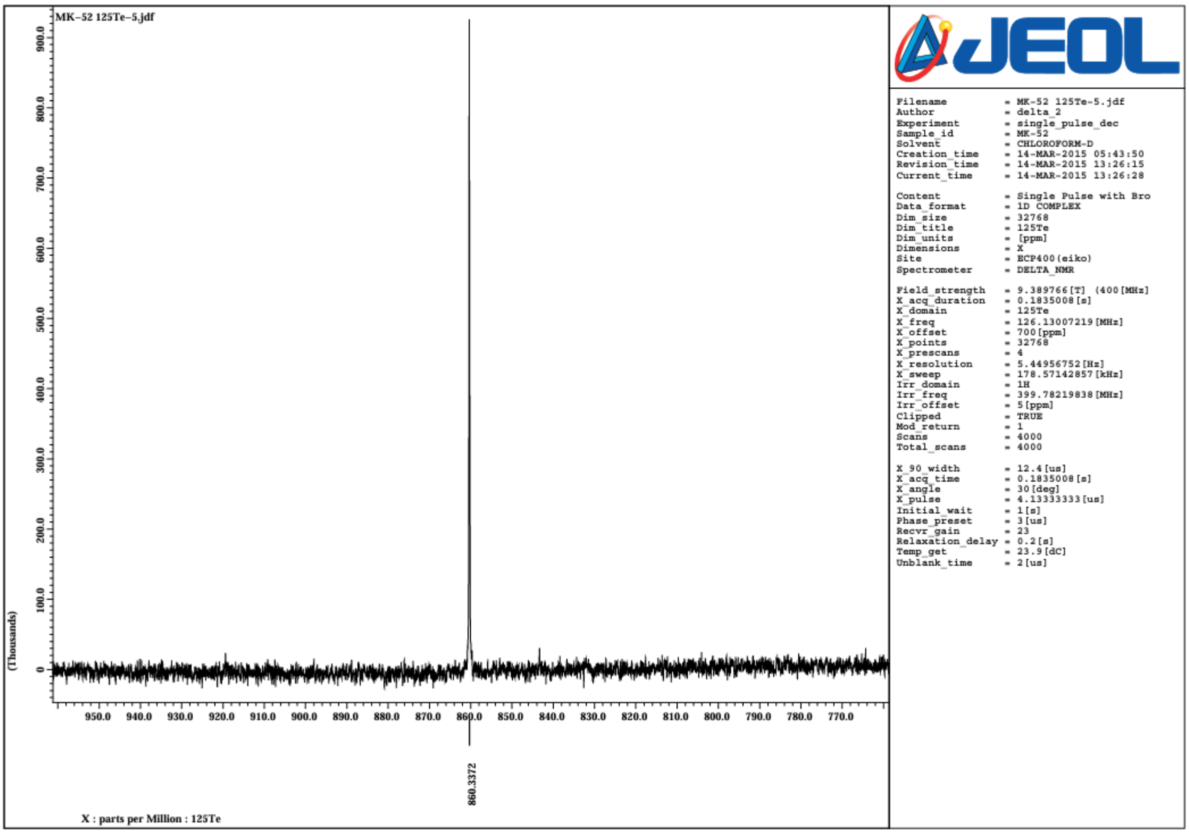


**Fig. S15.** $^{125}$Te NMR spectrum of compound Ph[PhC(OH)CHC(O)$CH_2$]$TeCl_2$ (**1**) in $CDCl_3$.

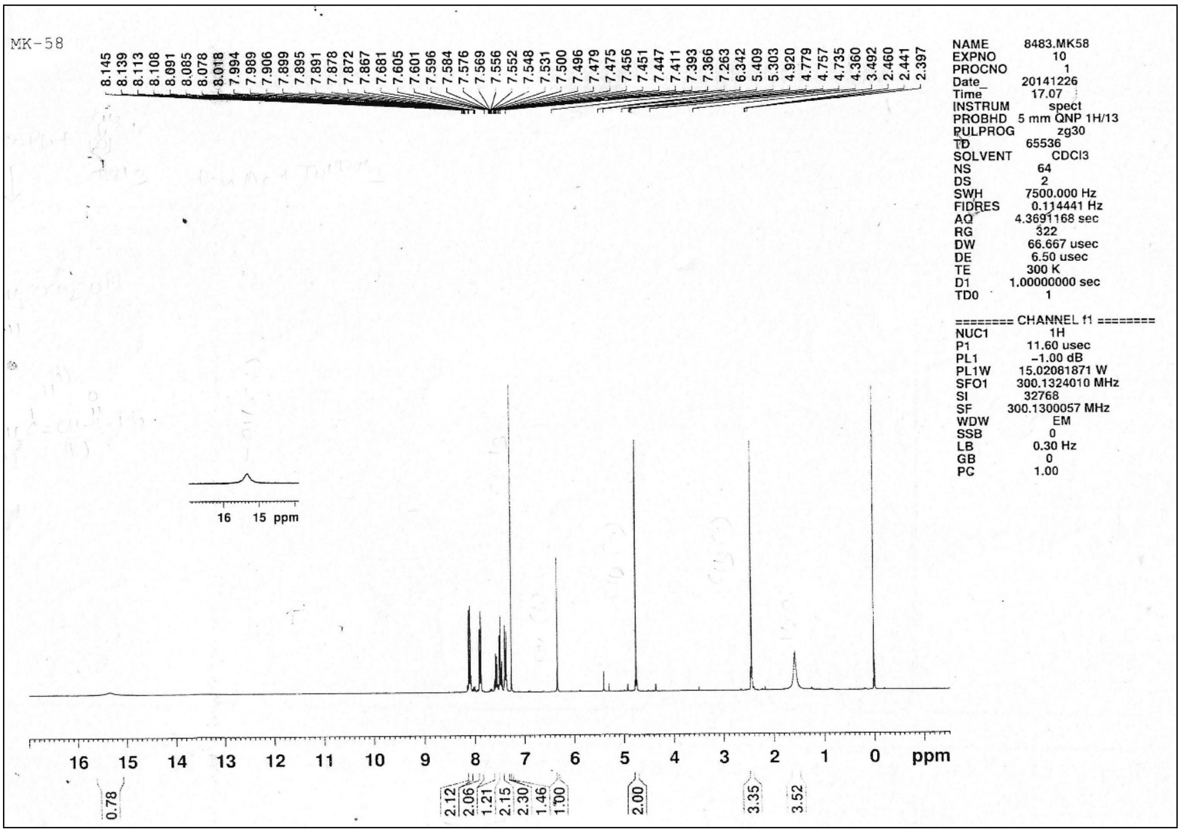


**Fig. S16.** $^{1}$H NMR spectrum of compound *p*-Tol[PhC(OH)CHC(O)$CH_2$]$TeCl_2$ (**2**) in $CDCl_3$.

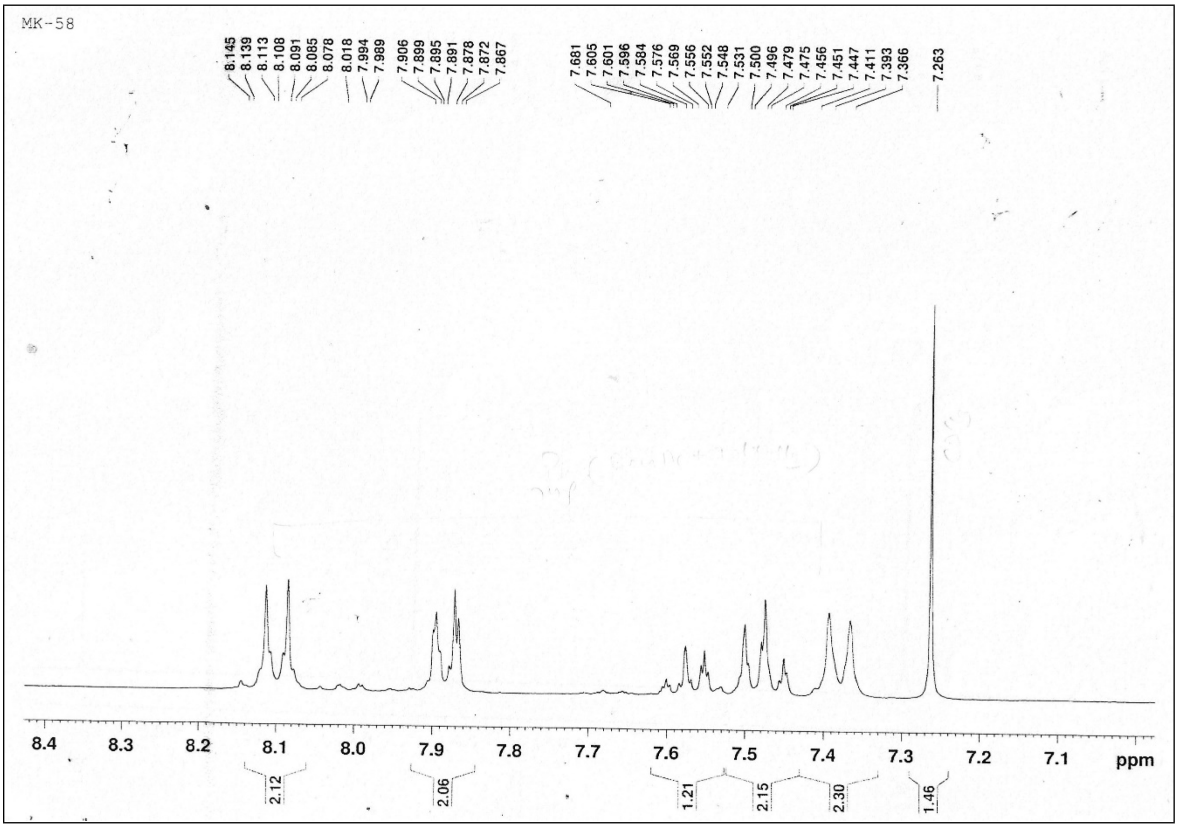


**Fig. S17.** Expanded aryl region of $^{1}$H NMR spectrum of compound *p*-Tol[PhC(OH)CHC(O)$CH_2$]$TeCl_2$ (**2**) in $CDCl_3$.

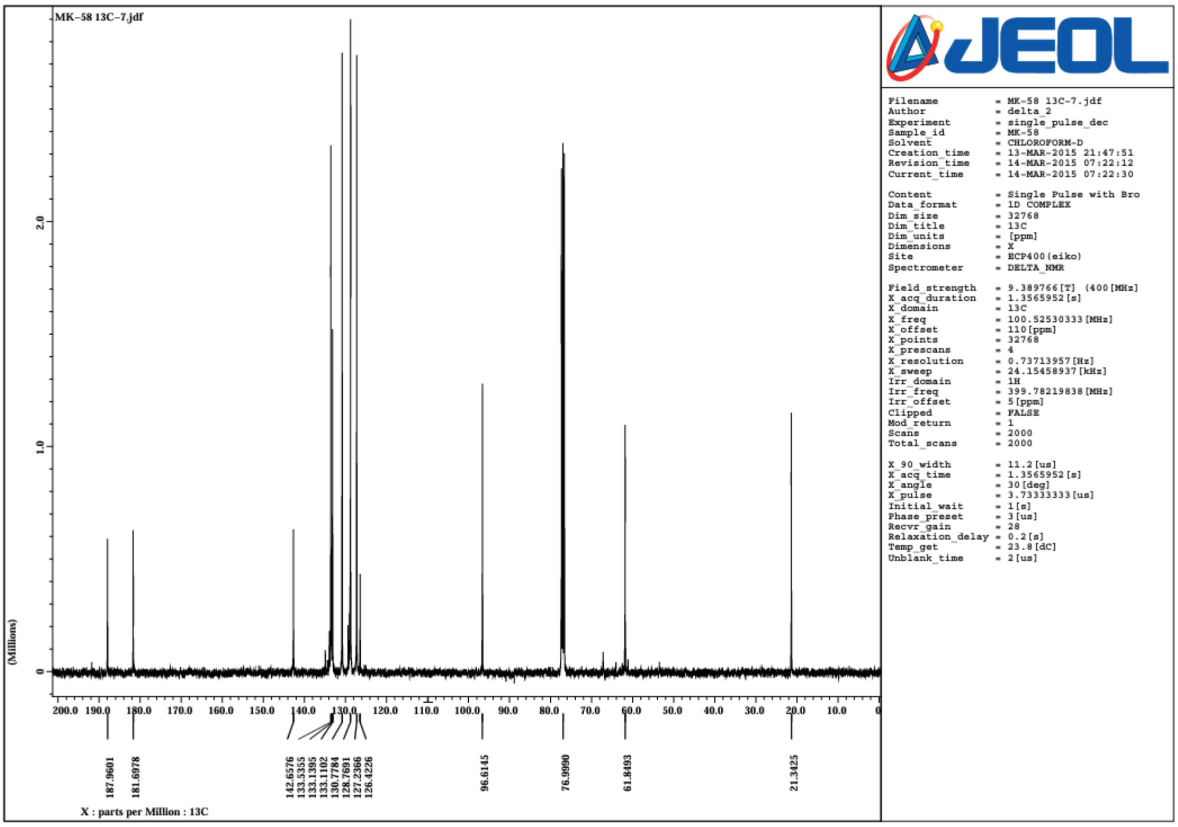


**Fig. S18.** $^{13}$C NMR spectrum of compound *p*-Tol[PhC(OH)CHC(O)$CH_2$]$TeCl_2$ (**2**) in $CDCl_3$.

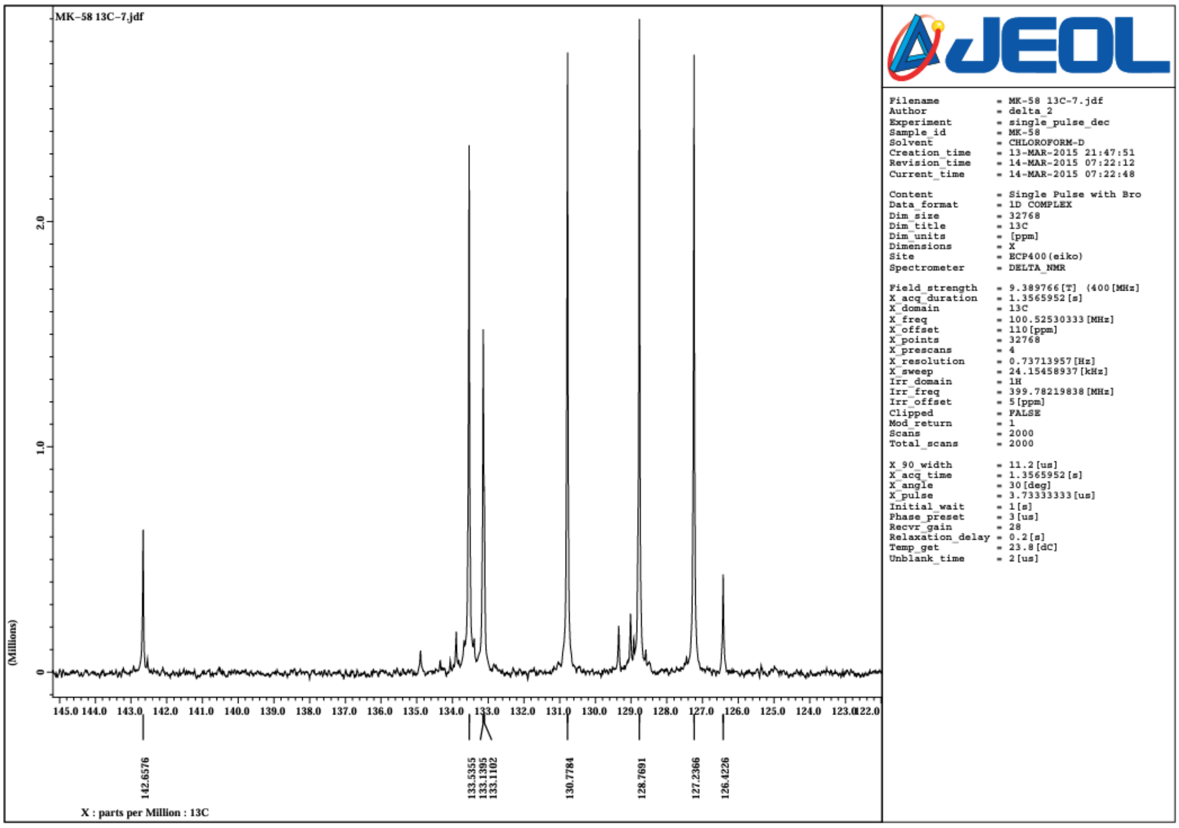


**Fig. S19.** Expanded aryl region of $^{13}C$ NMR spectrum of compound *p*-Tol[PhC(OH)CHC(O)$CH_2$]$TeCl_2$ (**2**) in $CDCl_3$.

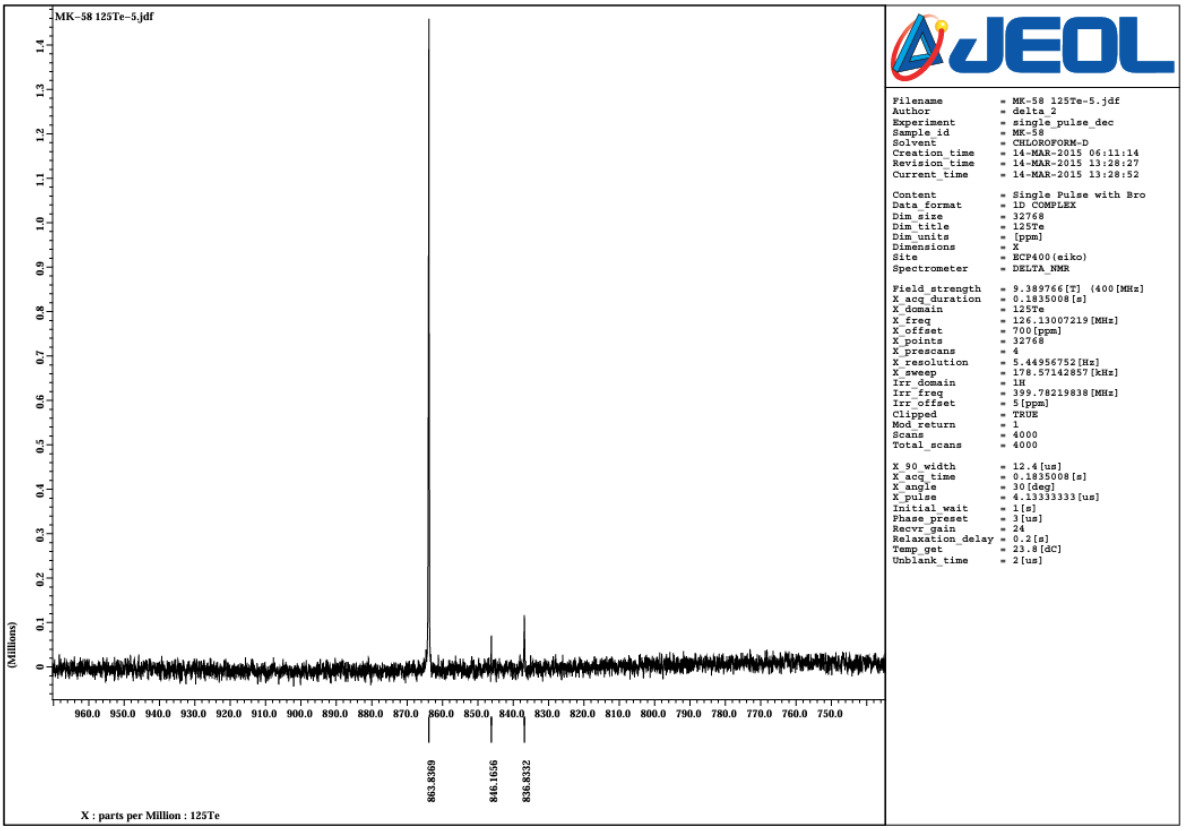


**Fig. S20.** $^{125}$Te NMR spectrum of compound *p*-Tol[PhC(OH)CHC(O)CH$_2$]TeCl$_2$ (**2**) in CDCl$_3$.

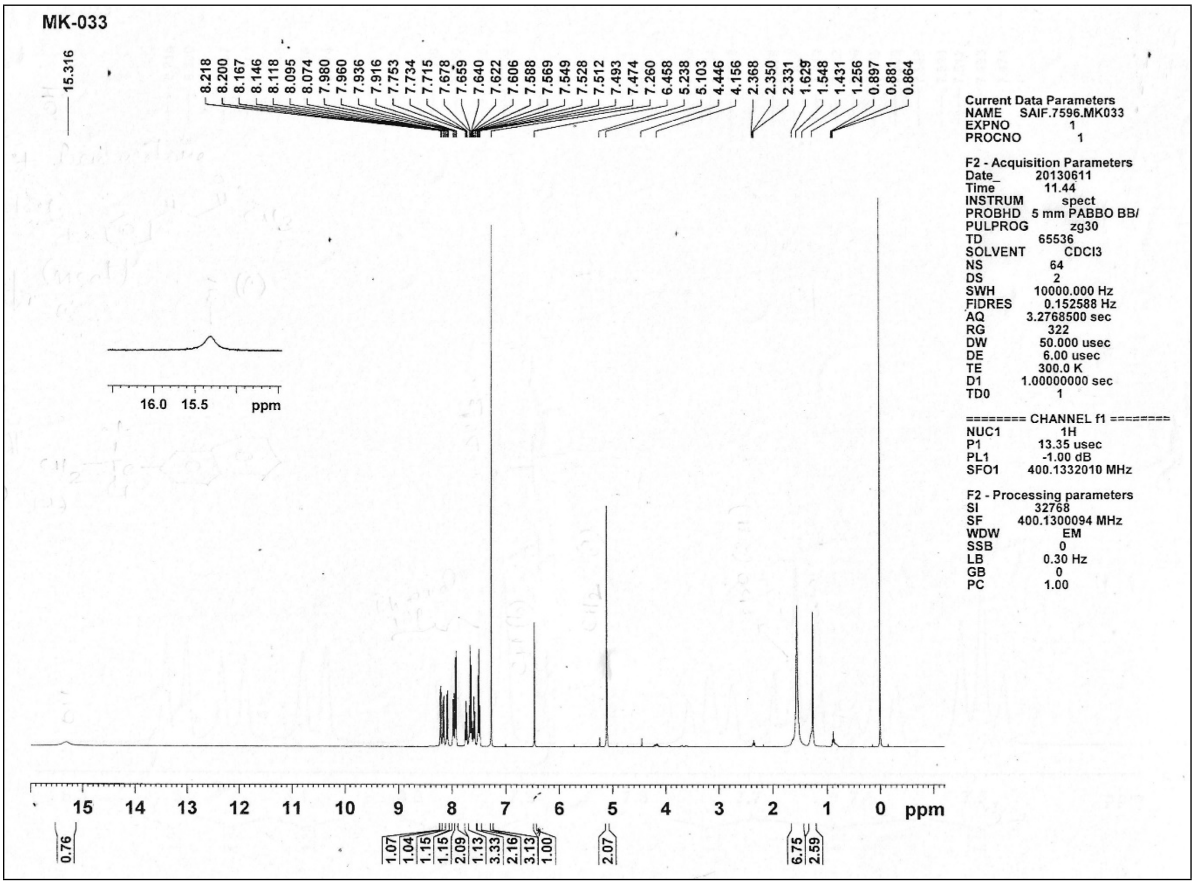


**Fig. S21.** $^{1}$H NMR spectrum of compound *1*-Nap[PhC(OH)CHC(O)$CH_2$]$TeCl_2$ (**3**) in $CDCl_3$.

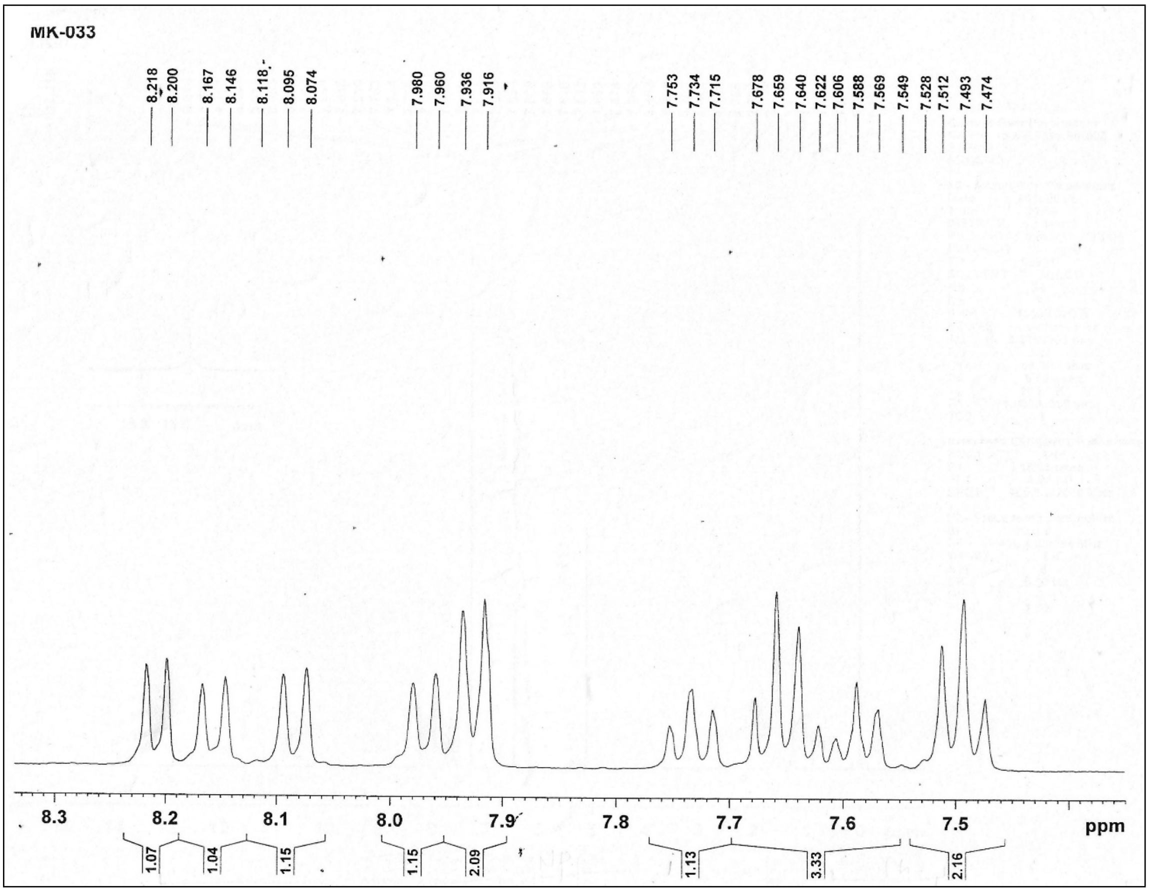


**Fig. S22.** Expanded aryl region of $^1$H NMR spectrum of compound *1*-Nap[PhC(OH)CHC(O)$CH_2$]$TeCl_2$ (**3**) in $CDCl_3$.

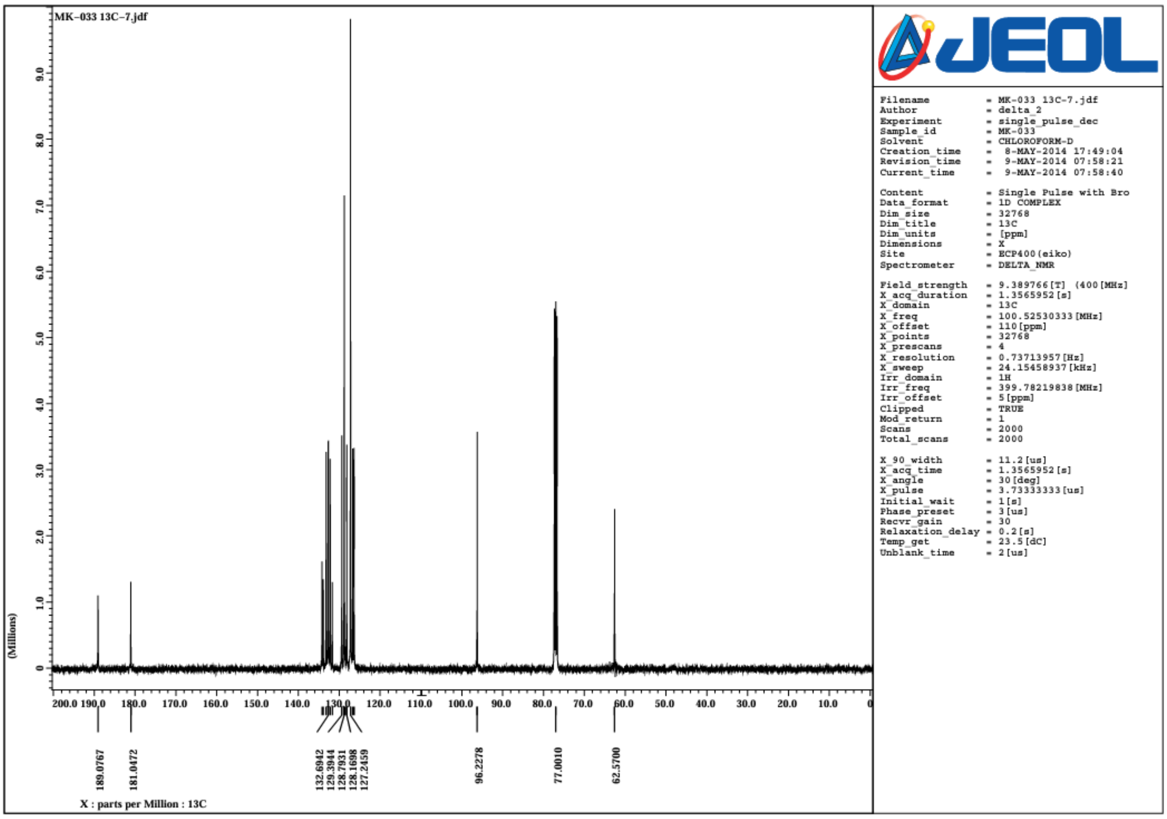


**Fig. S23.** $^{13}$C NMR spectrum of compound *1*-Nap[PhC(OH)CHC(O)$CH_2$]$TeCl_2$ (**3**) in $CDCl_3$.

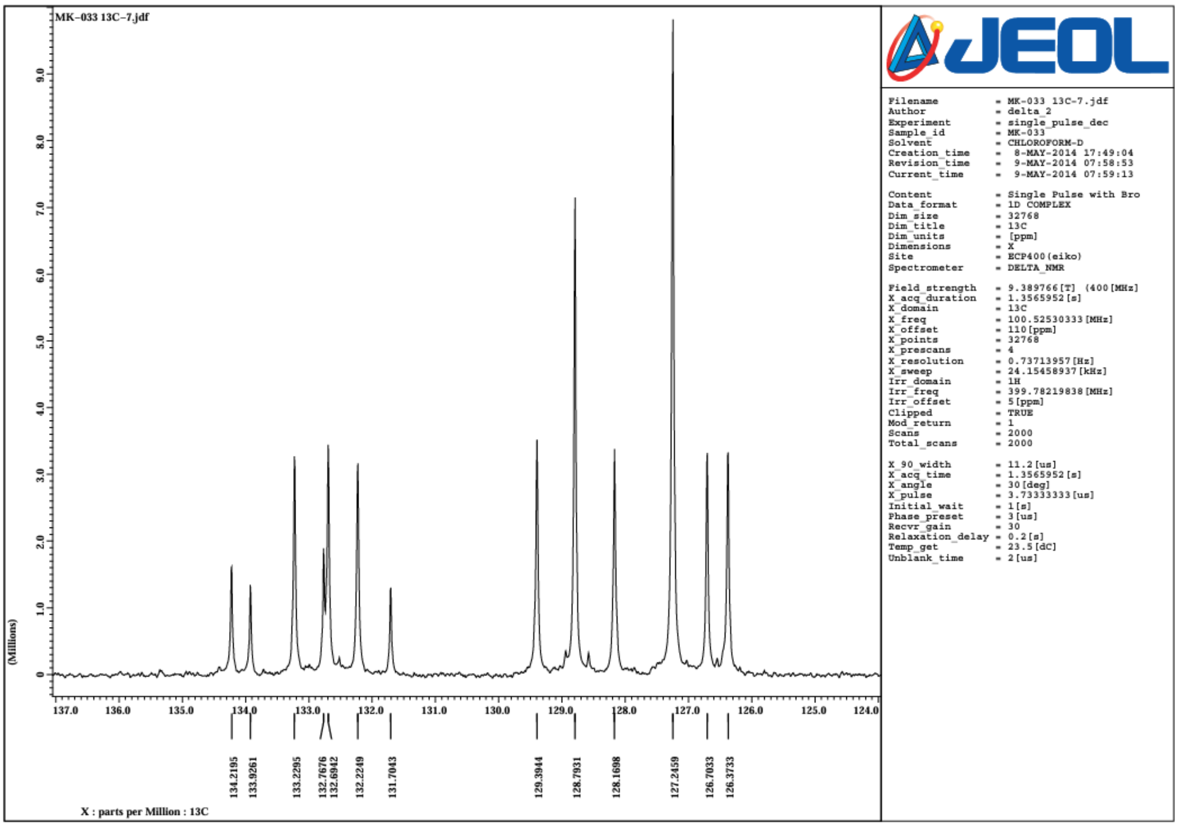


**Fig. S24.** Expanded aryl region of $^{13}C$ NMR spectrum of compound *1*-Nap[PhC(OH)CHC(O)$CH_2$]$TeCl_2$ (**3**) in $CDCl_3$.

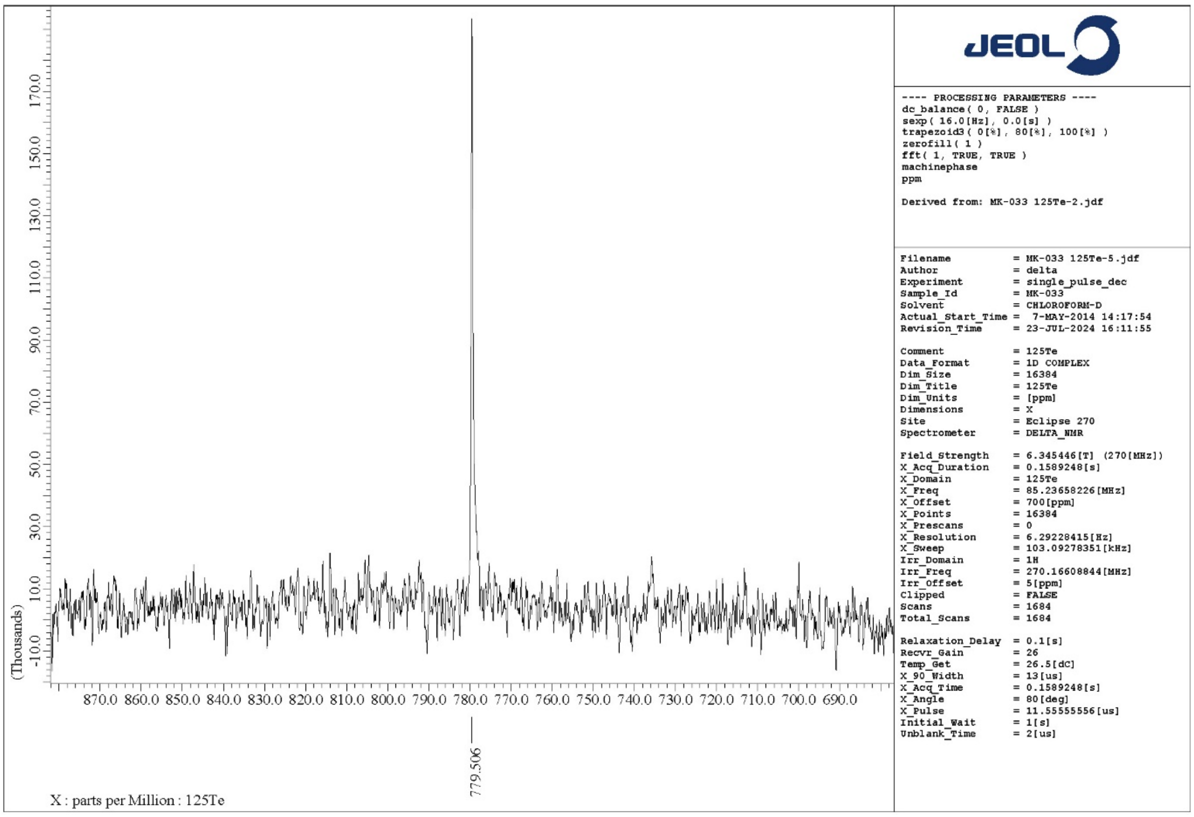


**Fig. S25.** $^{125}$Te NMR spectrum of compound *1*-Nap[PhC(OH)CHC(O)$CH_2$]$TeCl_2$ (**3**) in $CDCl_3$.

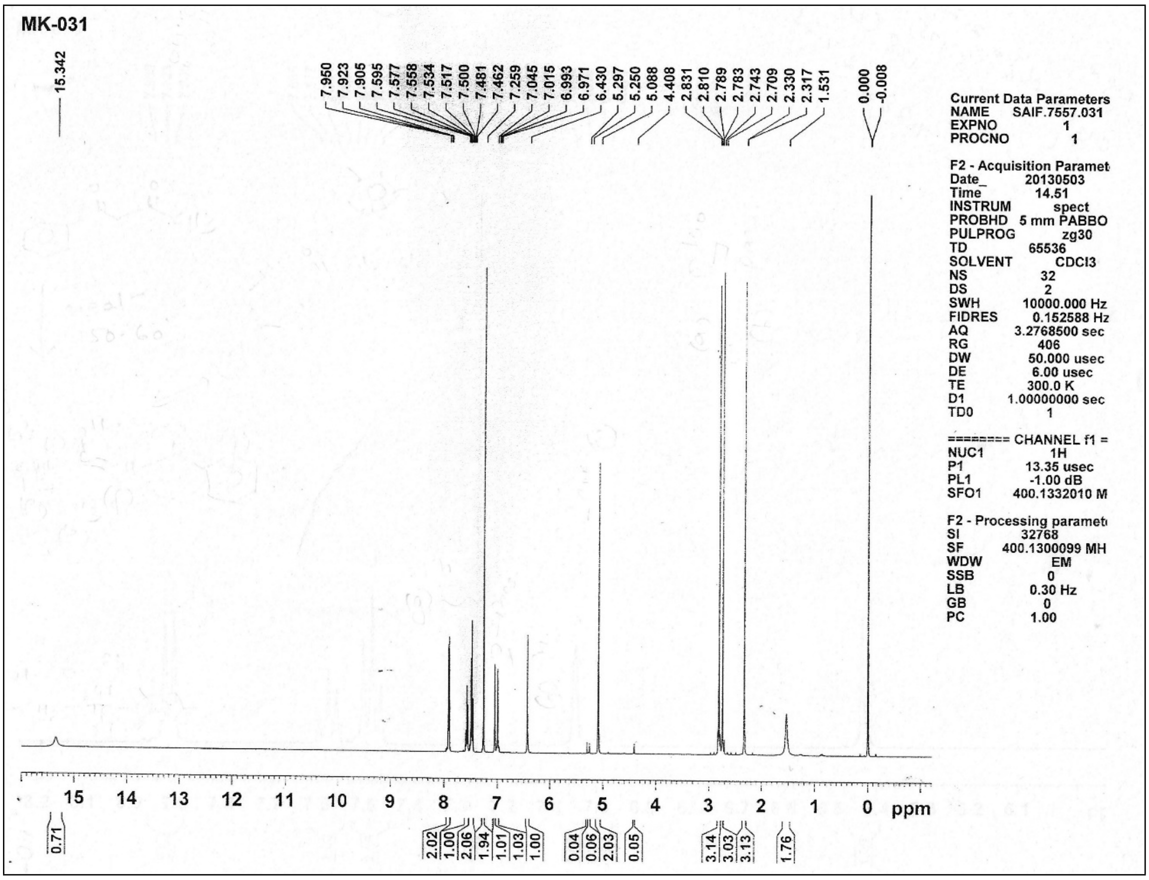


**Fig. S26.** $^{1}H$ NMR spectrum of compound Mes[PhC(OH)CHC(O)$CH_2$]$TeCl_2$ (**4**).

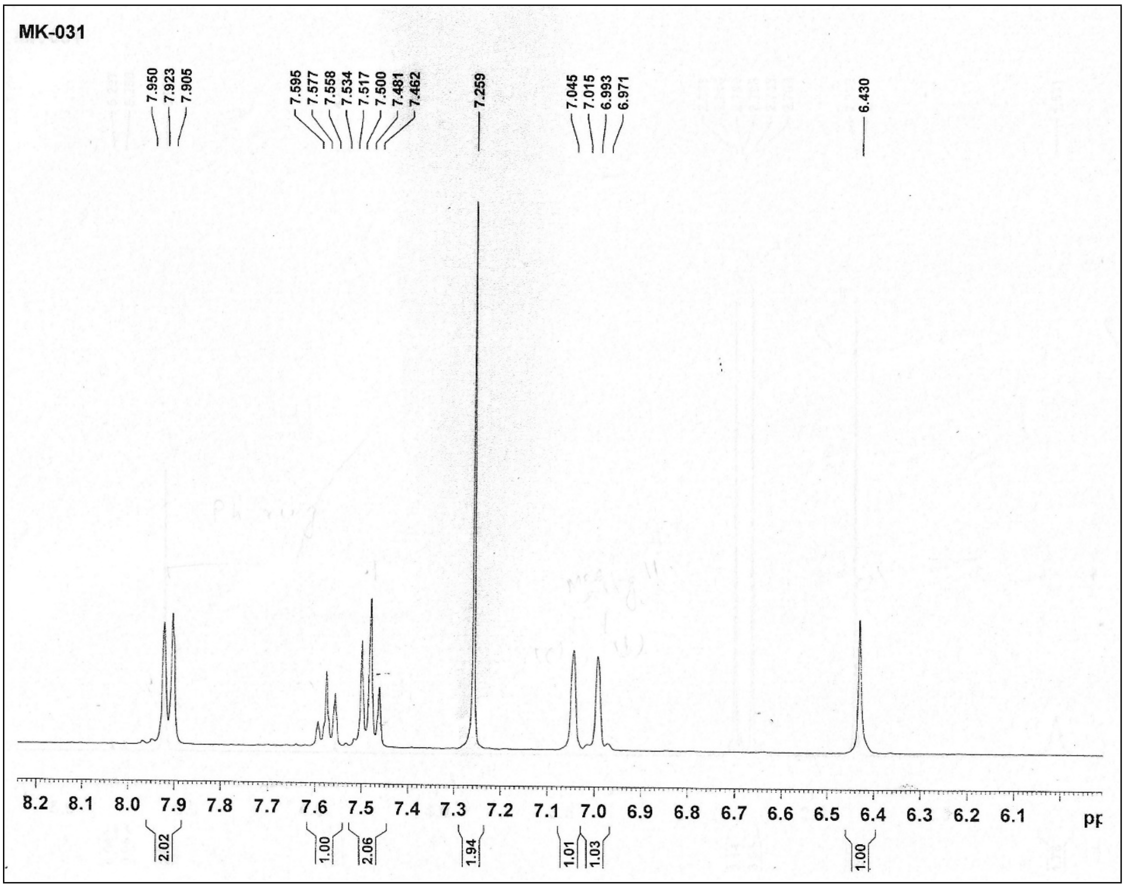


**Fig. S27.** Expanded aryl region of $^1$H NMR spectrum of compound Mes[PhC(OH)CHC(O)$CH_2$]$TeCl_2$ (**4**).

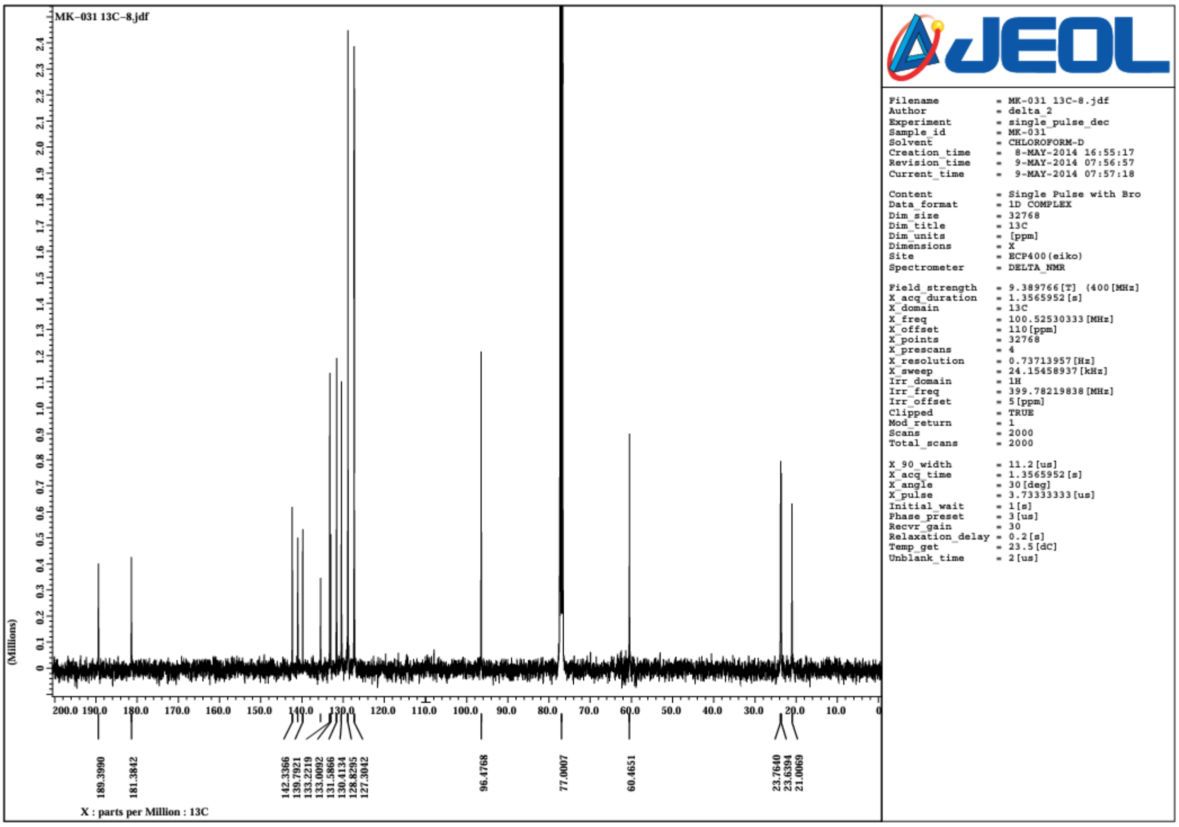


**Fig. S28.** $^{13}C$ NMR spectrum of compound Mes[PhC(OH)CHC(O)$CH_2$]$TeCl_2$ (**4**) in $CDCl_3$.

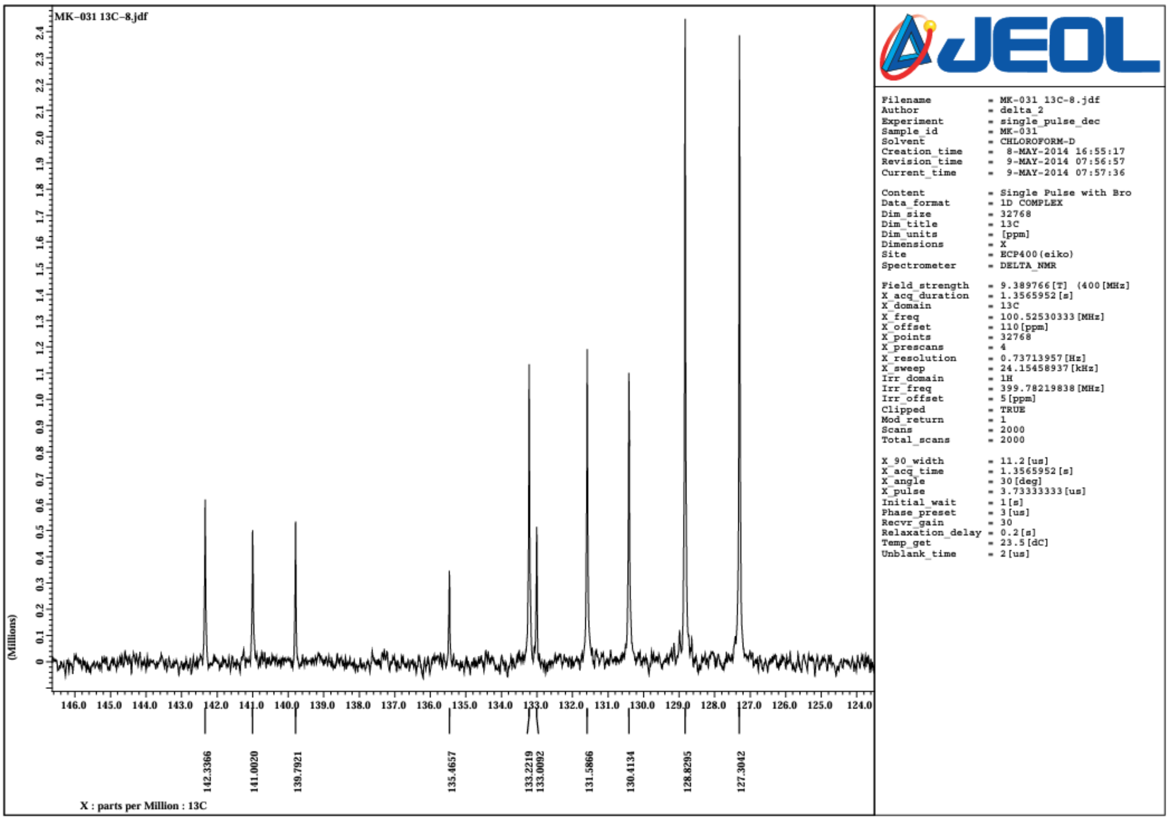


**Fig. S29.** Expanded aryl region of $^{13}C$ NMR spectrum of compound Mes[PhC(OH)CHC(O)$CH_2$]$TeCl_2$ (**4**) in $CDCl_3$.

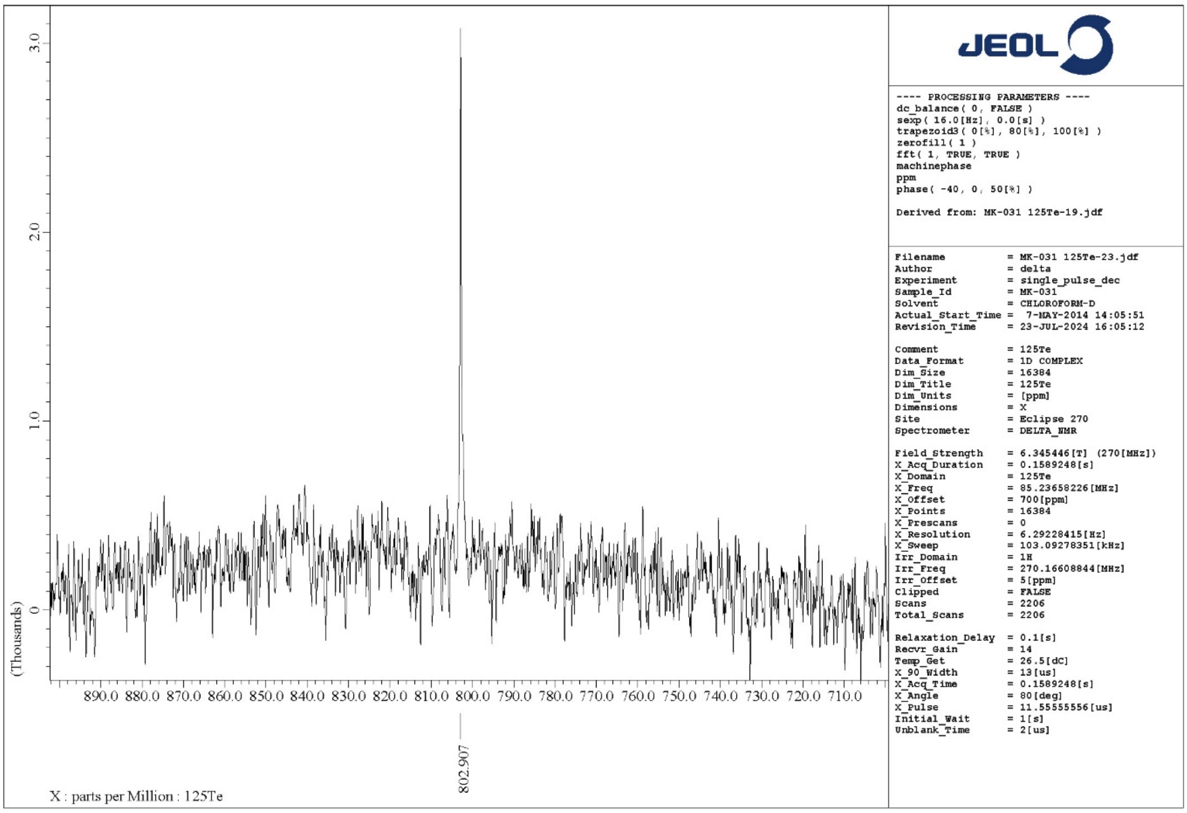


**Fig. S30.** $^{125}$Te NMR spectrum of compound Mes[PhC(OH)CHC(O)$CH_2$]$TeCl_2$ (**4**) in $CDCl_3$.

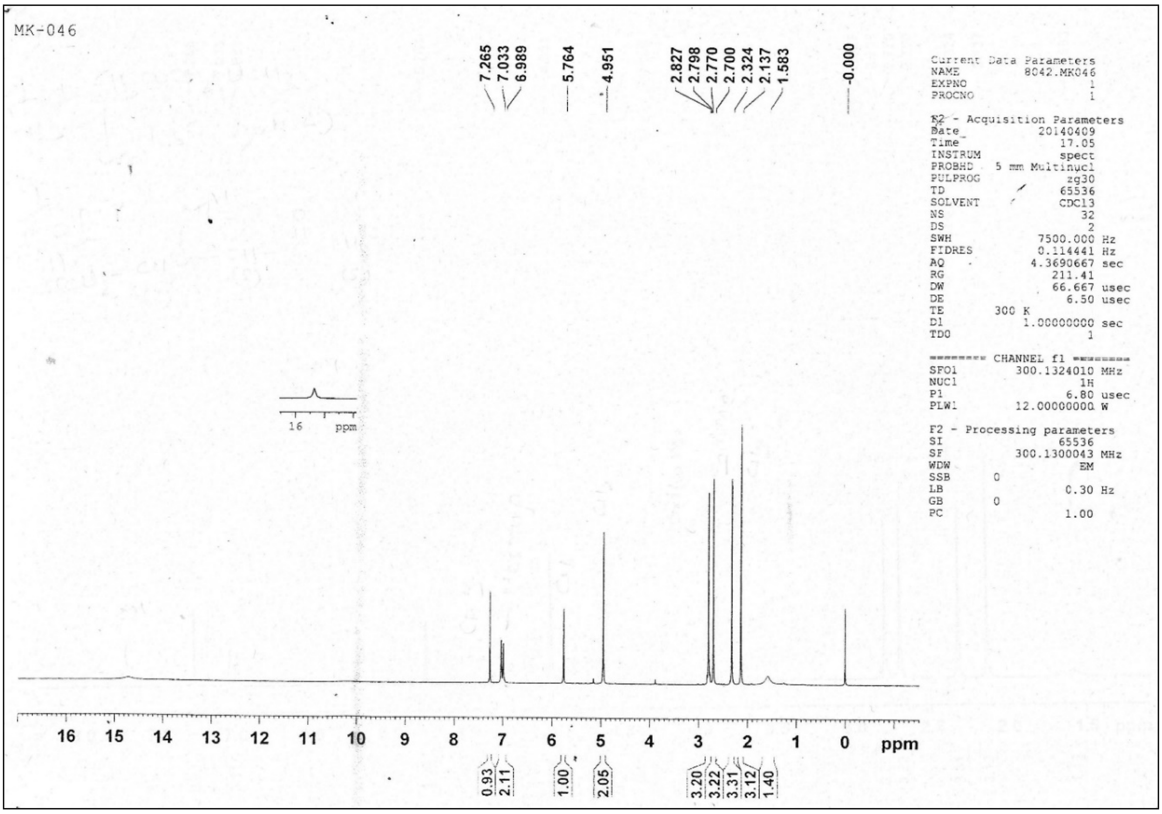


**Fig. S31.** $^{1}$H NMR spectrum of compound Mes[$CH_3$(OH)CHC(O)$CH_2$]$TeCl_2$ (**5**).

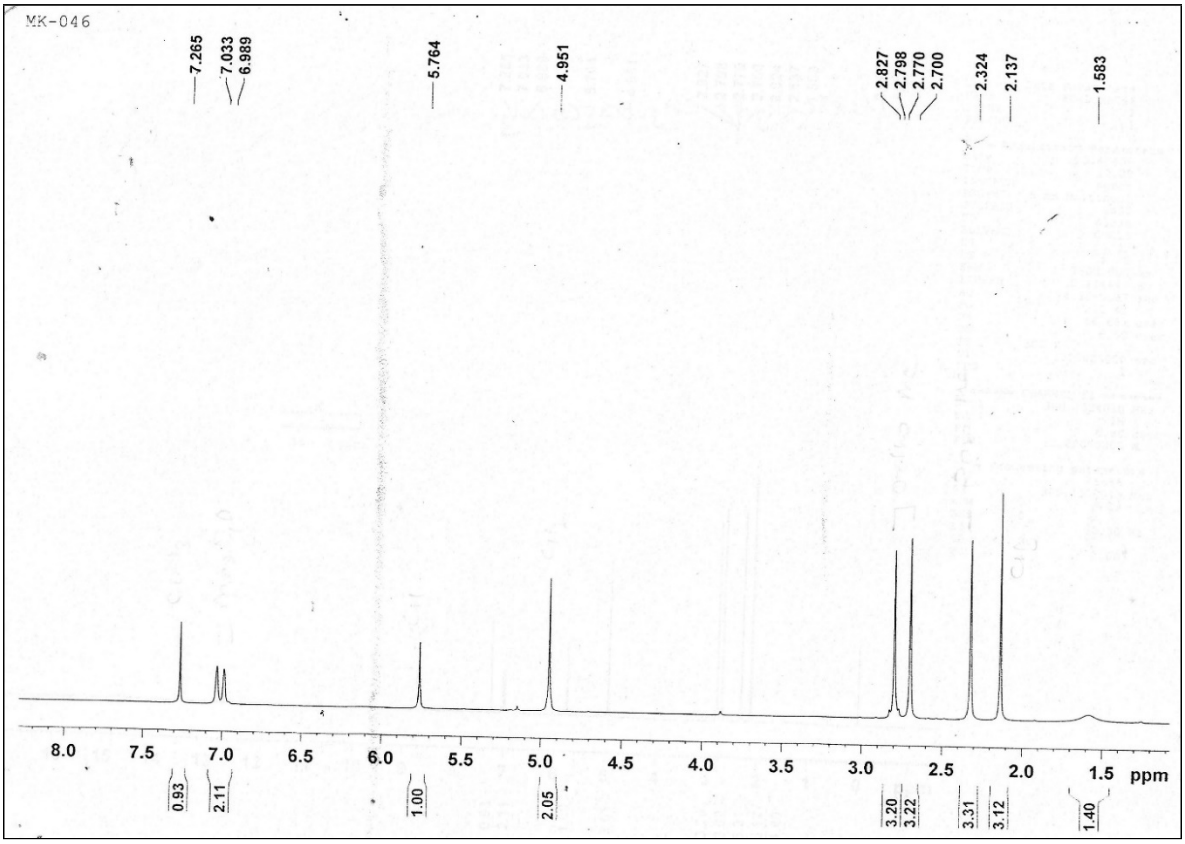


**Fig. S32.** Expanded aryl region of $^1$H NMR spectrum of compound Mes[$CH_3$(OH)CHC(O)$CH_2$]$TeCl_2$ (**5**).

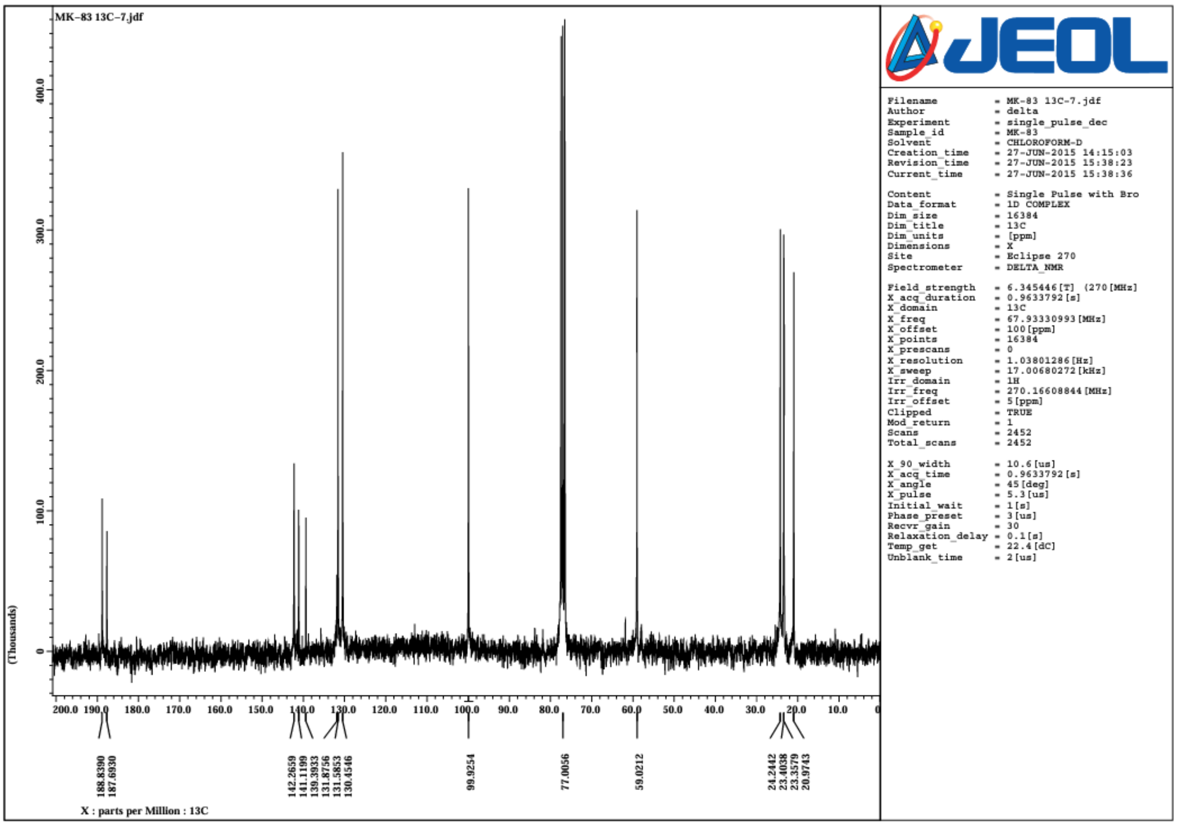


**Fig. S33.** $^{13}$C NMR spectrum of compound Mes[$CH_3$(OH)CHC(O)$CH_2$]$TeCl_2$ (**5**) in $CDCl_3$.

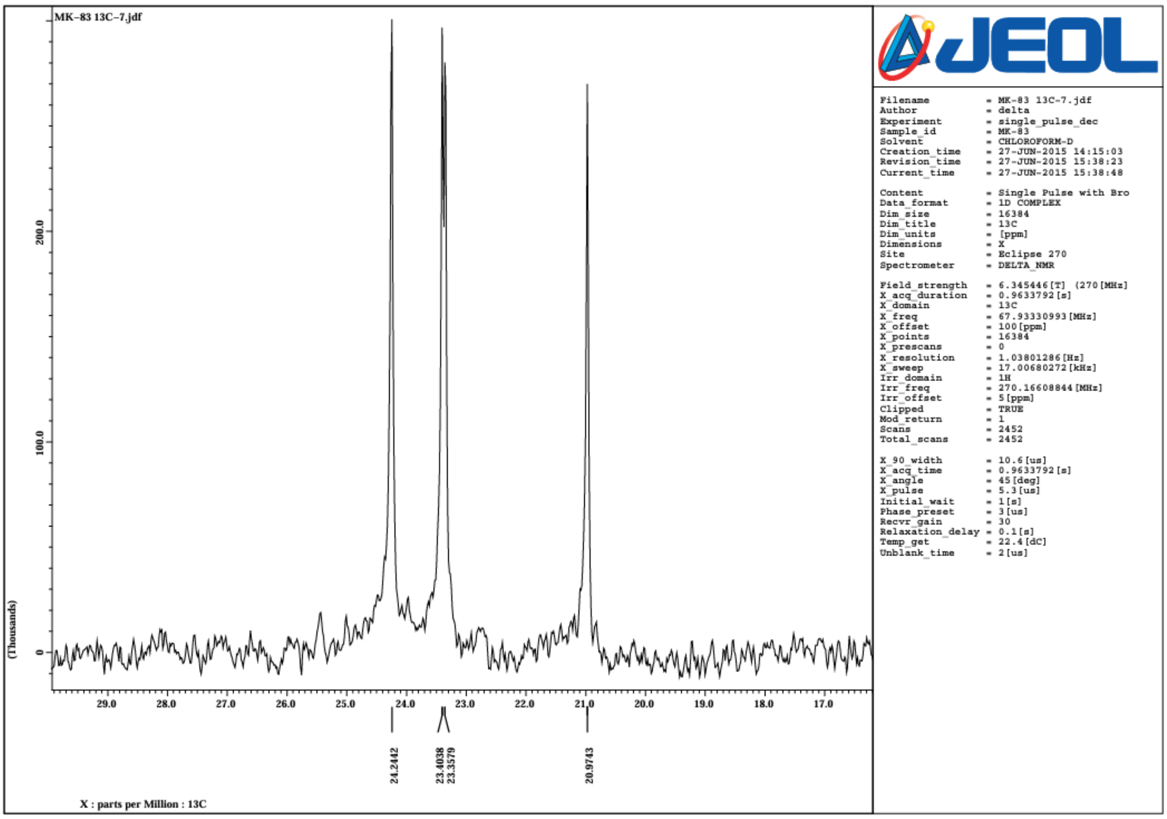


**Fig. S34.** Expanded alkyl region of $^{13}$C NMR spectrum of compound Mes[$CH_3$(OH)CHC(O)$CH_2$]$TeCl_2$ (**5**) in $CDCl_3$.

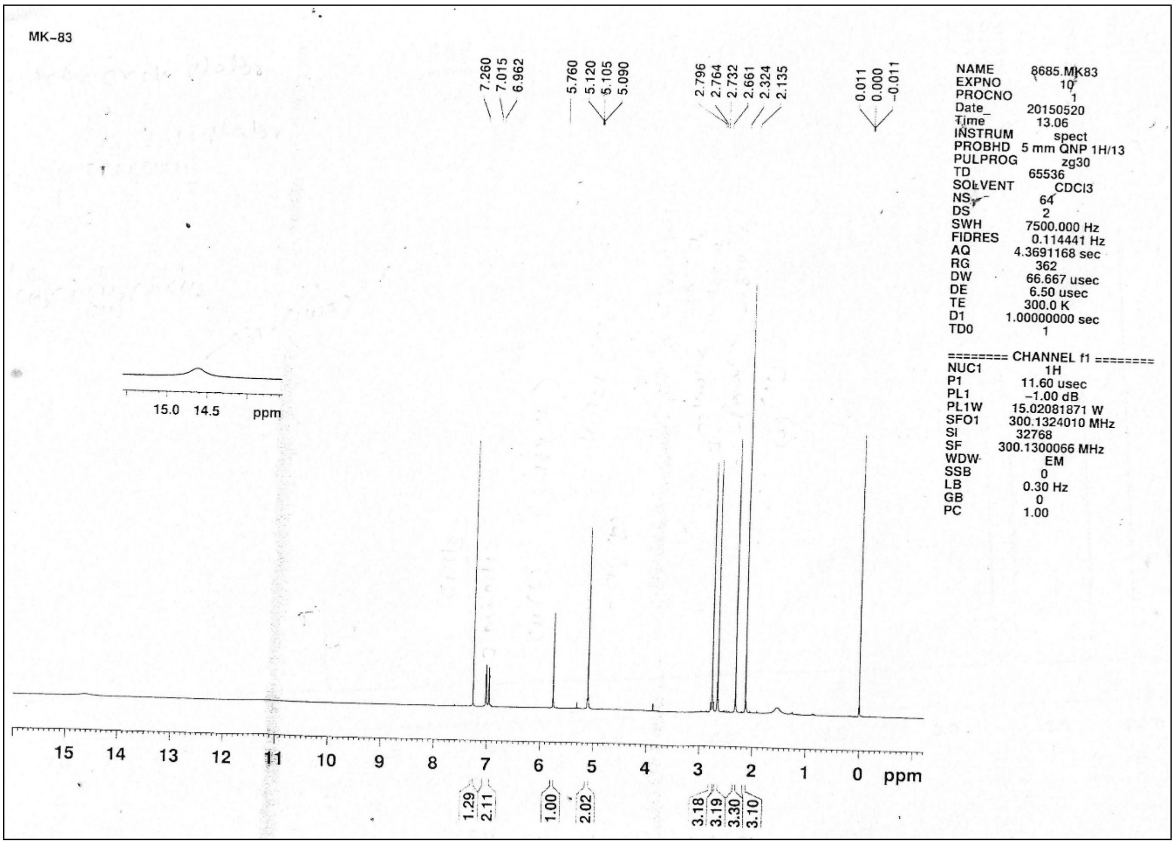

**Fig. S35.** $^{1}$H NMR spectrum of compound Mes[$CH_3$(OH)CHC(O)$CH_2$]$TeBr_2$ (**6**).

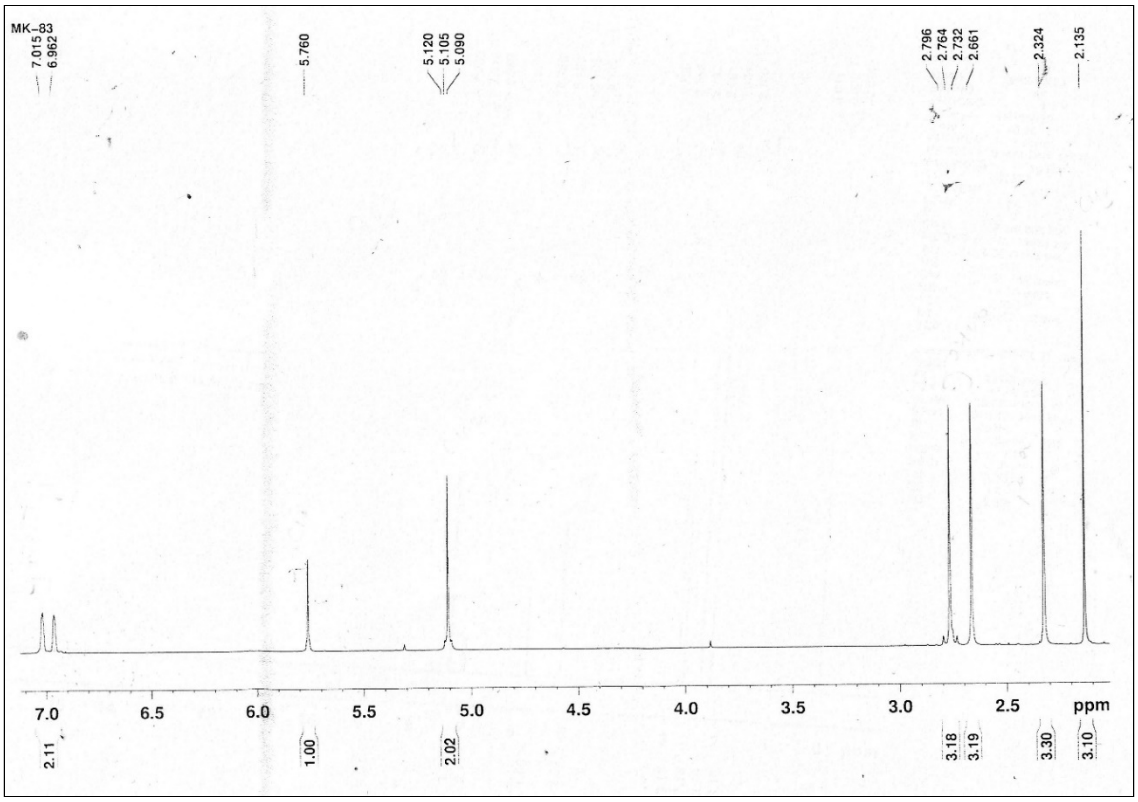


**Fig. S36.** $^{1}$H NMR spectrum of compound Mes[$CH_3$(OH)CHC(O)$CH_2$]$TeBr_2$ (**6**).

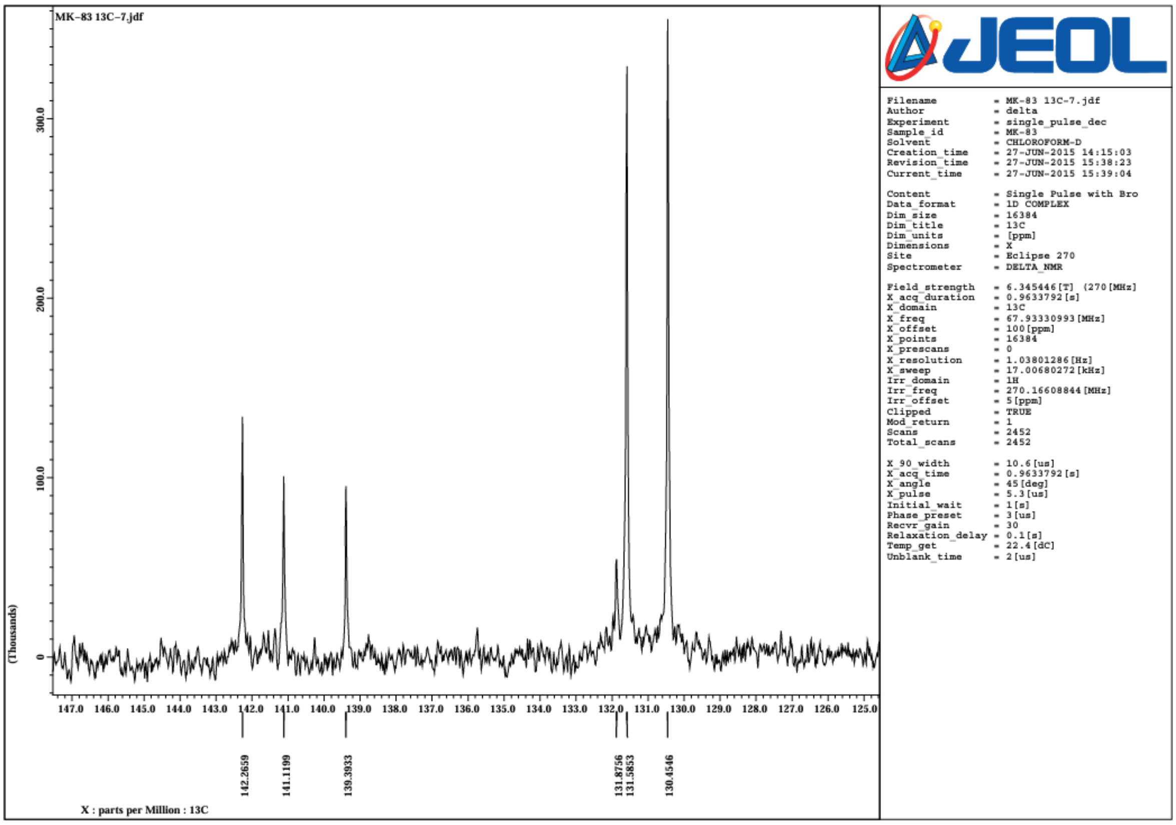


**Fig. S37.** Expanded aryl region of $^{13}C$ NMR spectrum of compound Mes[$CH_3$(OH)CHC(O)$CH_2$]$TeBr_2$ (**6**) in $CDCl_3$.

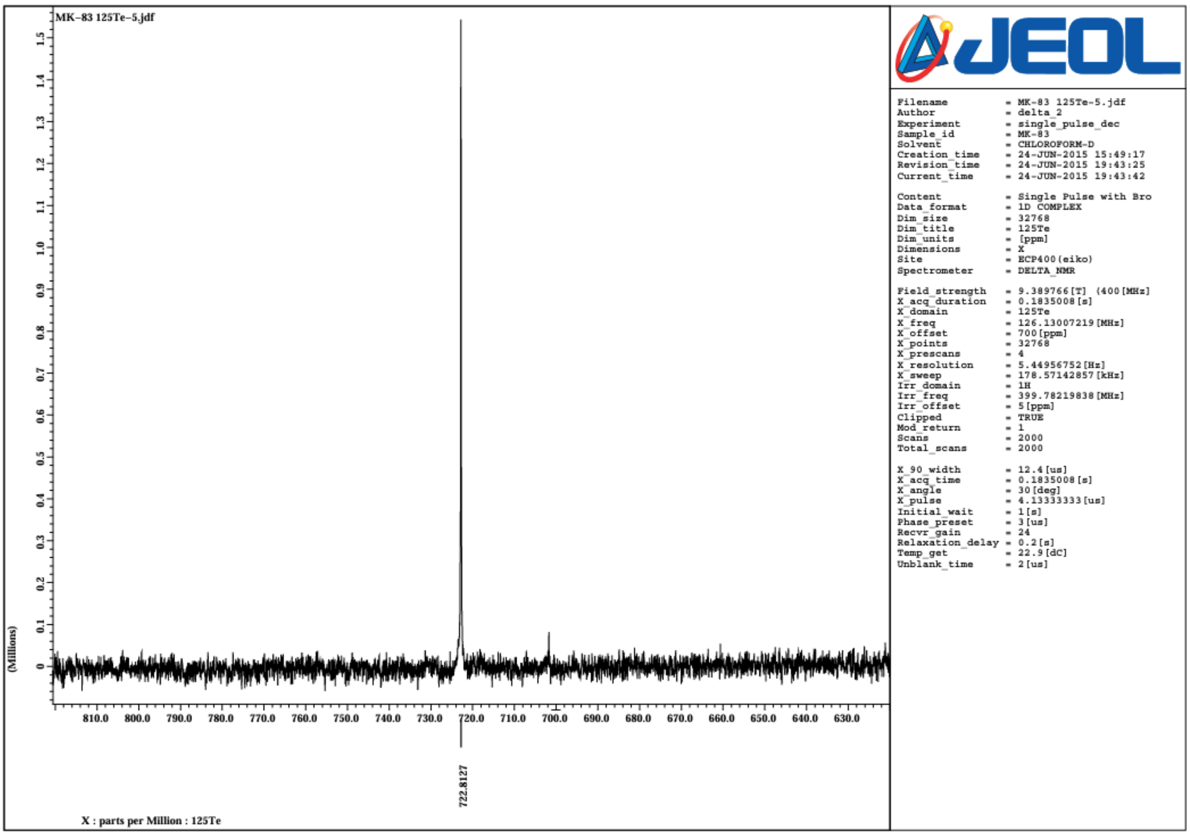


**Fig. S38.** $^{125}$Te NMR spectrum of compound Mes[$CH_3$(OH)CHC(O)$CH_2$]$TeBr_2$ (**6**) in $CDCl_3$.
